# Differential Selection on Carotenoid Biosynthesis Genes as a Function of Gene Position in the Metabolic Pathway: A Study on the Carrot and Dicots

**DOI:** 10.1371/journal.pone.0038724

**Published:** 2012-06-18

**Authors:** Jérémy Clotault, Didier Peltier, Vanessa Soufflet-Freslon, Mathilde Briard, Emmanuel Geoffriau

**Affiliations:** 1 Université d’Angers, UMR1345 Institut de Recherche en Horticulture et Semences, PRES L’UNAM, Angers, France; 2 Agrocampus Ouest, UMR1345 Institut de Recherche en Horticulture et Semences, Angers, France; 3 INRA, UMR1345 Institut de Recherche en Horticulture et Semences, Beaucouzé, France; Instituto de Biología Molecular y Celular de Plantas, Spain

## Abstract

**Background:**

Selection of genes involved in metabolic pathways could target them differently depending on the position of genes in the pathway and on their role in controlling metabolic fluxes. This hypothesis was tested in the carotenoid biosynthesis pathway using population genetics and phylogenetics.

**Methodology/Principal Findings:**

Evolutionary rates of seven genes distributed along the carotenoid biosynthesis pathway, *IPI*, *PDS*, *CRTISO*, *LCYB*, *LCYE*, *CHXE* and *ZEP*, were compared in seven dicot taxa. A survey of deviations from neutrality expectations at these genes was also undertaken in cultivated carrot (*Daucus carota* subsp. *sativus*), a species that has been intensely bred for carotenoid pattern diversification in its root during its cultivation history. Parts of sequences of these genes were obtained from 46 individuals representing a wide diversity of cultivated carrots. Downstream genes exhibited higher deviations from neutral expectations than upstream genes. Comparisons of synonymous and nonsynonymous substitution rates between genes among dicots revealed greater constraints on upstream genes than on downstream genes. An excess of intermediate frequency polymorphisms, high nucleotide diversity and/or high differentiation of *CRTISO*, *LCYB1* and *LCYE* in cultivated carrot suggest that balancing selection may have targeted genes acting centrally in the pathway.

**Conclusions/Significance:**

Our results are consistent with relaxed constraints on downstream genes and selection targeting the central enzymes of the carotenoid biosynthesis pathway during carrot breeding history.

## Introduction

One of the most important objectives of molecular evolution studies is to understand which factors influence genetic variations in the genome. Many genes are organized in signaling or metabolic pathways and are therefore related to protein-protein interactions or product-substrate relationships. Understanding how selection acts on genes involved in pathways or networks has received increasing attention in the study of molecular evolution in recent years [1], [2]. Two key factors were shown to be of particular relevance for explaining the evolution of metabolic pathways: node connectivity and the position of the gene in the pathway or network.

Enzymes acting directly downstream from metabolic nodes and therefore controlling metabolic allocation to subsequent metabolic branches are expected to experience more selective constraints than other enzymes in the pathway. Selection was thus found to be directed to genes encoding enzymes located at metabolic nodes in central metabolism in *Drosophila* [3] and starch pathway in maize [4].

Genes encoding upstream enzymes are expected to face stronger selective constraints and therefore to evolve more slowly than genes encoding downstream enzymes, maybe owing to differential pleiotropic effects [1]. Modeling showed that beneficial mutations are preferentially driven to upstream genes, and have a greater impact on flux control than downstream genes during adaptive evolution [2]. Neutral or slightly deleterious substitutions are more prone to be accumulated in downstream genes, with less control on metabolic fluxes [2]. These model predictions were confirmed by several empirical studies. In genes involved in several terpenoid pathways in plants, a correlation was evidenced between the ratio of nonsynonymous substitution to synonymous substitution rates (*ω* or *d_N_*/*d_S_*) and the position of genes along the pathway, suggesting progressive relaxation of selective constraints along metabolic pathways [5]. Slower evolution of upstream enzymes than downstream genes was also described in the anthocyanin biosynthetic pathway [6]–[8]. However, investigations of the phenylpropanoid pathway in *Arabidopsis thaliana* [9], of the gibberellin pathway in the *Oryzeae* tribe [10] and of the starch pathway in *Oryza sativa* [11] failed to provide evidence for a relation between the position of the genes in the pathway and selective constraints.

**Figure 1:**
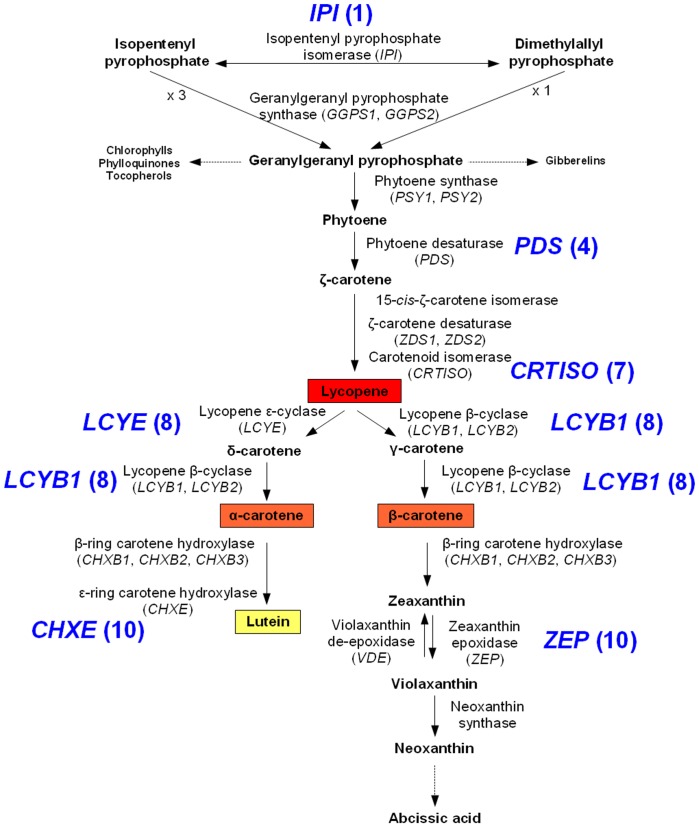
Carotenoid biosynthesis pathway in carrot. Names of genes in carrot, as described in [24], are in brackets. The name of genes used to search for signatures of selection is in bold, followed by the position number used to test the correlation between selection and pathway position. Boxes indicate main carotenoids found in carrot [17], [18].

The carotenoid biosynthesis pathway is also suitable network topology to investigate the effect of pathway position on gene evolution, as this pathway involves about ten enzymes acting at different positions and contains two metabolic nodes (Figure 1). Geranylgeranyl pyrophosphate (GGPP) is synthesized from isoprenoid precursors: isopentenyl pyrophosphate (IPP) and dimethylallyl pyrophosphate (DMAPP). GGPP is a main metabolic node since it is involved in the biosynthesis of chlorophylls, gibberellins, phylloquinones, plastoquinones, tocopherol and carotenoids [12]. The trunk of the carotenoid pathway involves the transformation of GGPP into lycopene. Lycopene is the direct precursor of two metabolic branches leading to lutein and abcissic acid respectively, and is thus the second node in this pathway.

Carotenoids act as accessory pigments and play a photoprotective role in the photosynthetic apparatus. They are also accumulated in large quantities in many fruits and flowers to attract animals required for pollination or seed dissemination [13]. Carotenoids are also involved in the wide range of colors observed in fruits, vegetables and ornamental plants. Therefore it could be expected that during plant domestication and plant improvement, some carotenoid biosynthesis genes were the target of natural or artificial selection. Stronger constraint on the upstream enzymes, phytoene desaturase (PDS), ζ-carotene desaturase (ZDS) and lycopene β-cyclase (LCYB), than on the downstream enzyme, zeaxanthin epoxidase (ZEP), was identified by analyzing *d_N_*/*d_S_* ratio in six dicots [14]. The gene *Y1* encoding the upstream enzyme PSY has experienced positive selection during the evolution of grasses [15] and maize modern breeding for yellow kernels [16]. Except for these examples, very few authors have investigated selection pressures on genes involved in the carotenoid biosynthesis pathway.

Carrot (*Daucus carota* L. ssp. *sativus*) is a good model for such a study as this species exhibits a range of root colors that mainly depend on variable carotenoid profiles, except for the purple type, which is colored by anthocyanins [17], [18]. This color variability results from plant breeding activities during the history of cultivation of this species [19], [20]. The domestication of carrot is thought to have occurred in Afghanistan around 900 AD [21]. The first cultivated carrots had purple or yellow roots. White and orange colored carrots were first described in Western Europe in the early 17^th^ century [19]. Red carrots appeared in China and India in the 18^th^ century [22], [23]. According to this history, it makes sense to consider that carotenoid biosynthesis genes may have been targeted by artificial selection for color in carrot. The recent cloning of most of the carotenoid pathway genes in carrot offers the opportunity to investigate signatures of selection in this pathway [24].

The aim of this study was to investigate the pattern of signatures of selection in the carotenoid biosynthesis pathway and to check whether selection has been influenced by the position of the gene in this metabolic pathway. We used a population genetics approach to test for departures from neutral expectations at seven genes distributed along the carotenoid biosynthesis pathway in carrots with different colored roots. We then used a phylogenetics approach to test the same genes for variations in evolutionary rates in dicots. A signature of balancing selection was detected in genes around the metabolic node lycopene, in carrot. A significant shift toward lower neutrality test p-values was found for downstream genes by comparison with upstream genes. The phylogenetic analysis revealed greater constraints on upstream genes than on downstream genes.

## Results

This study aimed at testing a first hypothesis: downstream genes in the carotenoid biosynthesis pathway are less constrained than upstream genes. If this hypothesis is true, upstream genes must show lower *d_N_*/*d_S_* ratios than downstream genes in the phylogenetic analyses. In population genetic analyses, we would expect more deviations to neutral expectations for downstream genes than upstream genes, because of a relaxation of purifying selection in downstream genes and therefore a higher propensity to exhibit positive or balancing selection. The second hypothesis we examined is that the selection on carotenoid biosynthesis genes is most pronounced at pathway nodes. If this hypothesis is true, we would expect more deviations to neutral expectations in genes near the two pathway nodes phytoene and lycopene.

### Relationship between Nucleotide Patterns and Pathway Position

To test the relationship between the nucleotide variation and the position of genes in carotenoid biosynthesis pathway in carrot, we first checked for heterogeneity in the results of the neutrality tests performed on Tajima’s *D*, Fay and Wu’s *H* and *F_ST_* statistics between genes. The location parameters of the distribution of neutrality test p-values were not the same for each gene (Kruskal-Wallis rank sum test, *P* = 0.007). Therefore, the results of the neutrality tests were not equal between the seven genes.

**Figure 2:**
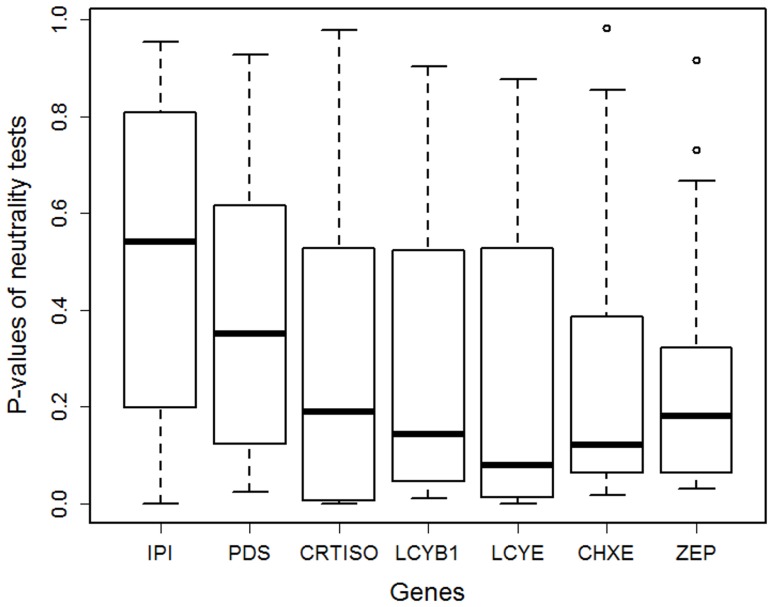
Selection in carotenoid biosynthesis pathway in carrot as a function of gene position. Distribution of p-values obtained according the rank of Tajima’s *D*, normalized Fay and Wu’s *H* and *F_ST_* for the seven carotenoid biosynthesis genes in carrot by comparison with the expected distribution obtained by approximate Bayesian computation simulations under the divergence model. Each boxplot combines p-values obtained on pooled, geographic and color samples. The genes are sorted according to their pathway position.

The two genes located upstream in the pathway, *IPI* and *PDS*, showed the biggest trend toward highest p-values (Figure 2). Genes located upstream from lycopene (*IPI*, *PDS*, *CRTISO*) had higher p-values than genes located downstream from lycopene (*LCYB1*, *LCYE*, *CHXE*, *ZEP*) (Wilcoxon rank sum test, *P*<0.004). P-values associated with the three tests taken globally correlated negatively with pathway position (Kendall’s correlation test: *τ* = −0.15; *P* = 0.004). Considering the three tests individually, only p-values associated with *F_ST_* showed a significant correlation with pathway position (*τ* = −0.18; *P* = 0.02). These results showed that polymorphism patterns in downstream genes deviated more from neutral expectations than those of upstream genes.

**Figure 3:**
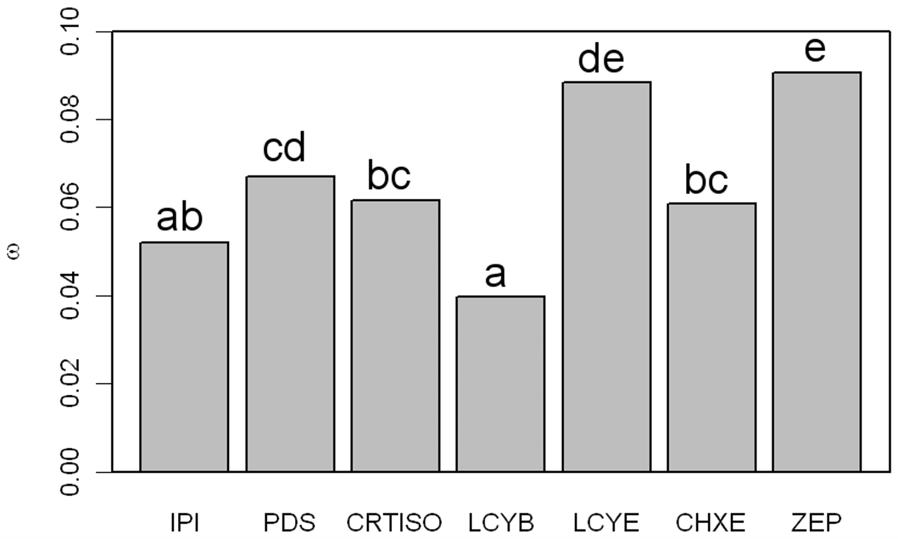
Values of dN/dS estimated by the M0 model for each carotenoid biosynthesis gene. Orthologs retrieved from seven dicots were used for this analysis. The genes are sorted according to their pathway position. The letters above each bar give the groups of significance according to the method described in [7].

In order to test if this observation is specific to carrot or could be extended to other species, we tested the carotenoid biosynthesis genes for variations in evolutionary rates (*d_N_*/*d_S_* = *ω*) in dicots. According to the M0 model, which assumes a constant *ω* in all branches and all codons, the estimated *ω* ratio varied from 0.040 in *LCYB* to 0.091 in *ZEP* (Table 1; Figure 3). To test the significance of the *ω* ratio variations among genes, we compared the likelihood obtained for the M0 model and for models assuming a constrained *ω* intermediate between the *ω* values estimated by the M0 model for each gene being compared (Figure 3). The model M0 applied to *IPI* and *LCYB* did not fit any better than the same model when *ω* was constrained to 0.046 (P>0.05), indicating that the *d_N_*/*d_S_* of these two genes was not significantly different. Similar results were obtained with comparisons of *ω* between *IPI*, *CRTISO* and *CHXE* (constrained *ω* tested = 0.053), between *PDS*, *CRTISO* and *CHXE* (constrained *ω* tested = 0.064), between *PDS* and *LCYE* (constrained *ω* tested = 0.078), and between *LCYE* and *ZEP* (constrained *ω* tested = 0.089). The groups of significance are summarized in Figure 3. The lowest *ω* values were obtained for *LCYB* and *IPI*, while the highest values were obtained for *LCYE* and *ZEP*.

**Table 1:** Parameter estimates and tests of selection for phylogenetic analysis of variation in the *ω* = *d_N_*/*d_S_* ratio in the carotenoid biosynthesis pathway.

	M0		M1		M2				M1a			
Gene	LnL	ω	LnL	p(χ^2^)	LnL	ω_1_	ω_0_	p(χ^2^)	LnL	ω_0_	p_0_	p(χ^2^)
***IPI***	−2535	0.052	−2532	0.759	−2535	0.053	0.049	0.815	−**2514**	0.038	0.944	**0.000** ***
***PDS***	−5658	0.067	−**5645**	**0.004** **	−5657	0.094	0.062	0.058	−**5594**	0.038	0.914	**0.000** ***
***CRTISO***	−5882	0.062	−5877	0.478	−5882	0.065	0.061	0.779	−**5830**	0.037	0.922	**0.000** ***
***LCYB***	−5243	0.040	−5236	0.143	−5243	0.037	0.040	0.716	−**5181**	0.025	0.936	**0.000** ***
***LCYE***	−4884	0.088	−4876	0.084	−4884	0.099	0.086	0.561	−**4789**	0.043	0.876	**0.000** ***
***CHXE***	−6189	0.061	−**6177**	**0.007** **	−6188	0.086	0.058	0.107	−**6136**	0.044	0.937	**0.000** ***
***ZEP***	−7114	0.091	−**7094**	**0.000** ***	−7114	0.093	0.090	0.900	−**7003**	0.051	0.869	**0.000** ***

M0 is a model that assumes a constant *ω* ratio for all phylogenetic branches and all codons. M1 and M2 are branch models that assume variations in the *ω* ratio in the phylogeny, but consider a constant *ω* ratio for all codons. M1 assumes an independent *ω* ratio for each branch. M2 assumes a specific *ω*_1_ ratio for the carrot branch, in comparison with the background *ω*_0_ ratio of the remaining branches. M1a and M2a are site models that assume different classes of codons with contrasting *ω* ratios, but a constant *ω* ratio in the phylogeny. M1a assumes two different classes of codons: codons with 0<*ω_0_*<1 at a frequency *p_0_* and other codons with *ω_1_* = 1 at a frequency *p_1_*. M2a assumes three classes of codons: codons with 0<*ω_0_*<1 at a frequency *p_0_*, *ω* = 1 at a frequency *p_1_* and *ω_2_*>1 at a frequency *p_2_*_._ We did not detect any codons in the latter class and therefore did not display results for M2a. The likelihood (LnL) is shown for each model, with the p-value p(χ^2^) associated with the likelihood ratio test.

* : *P*<0.05;

** : *P*<0.01;

*** : *P*<0.001.

**Figure 4:**
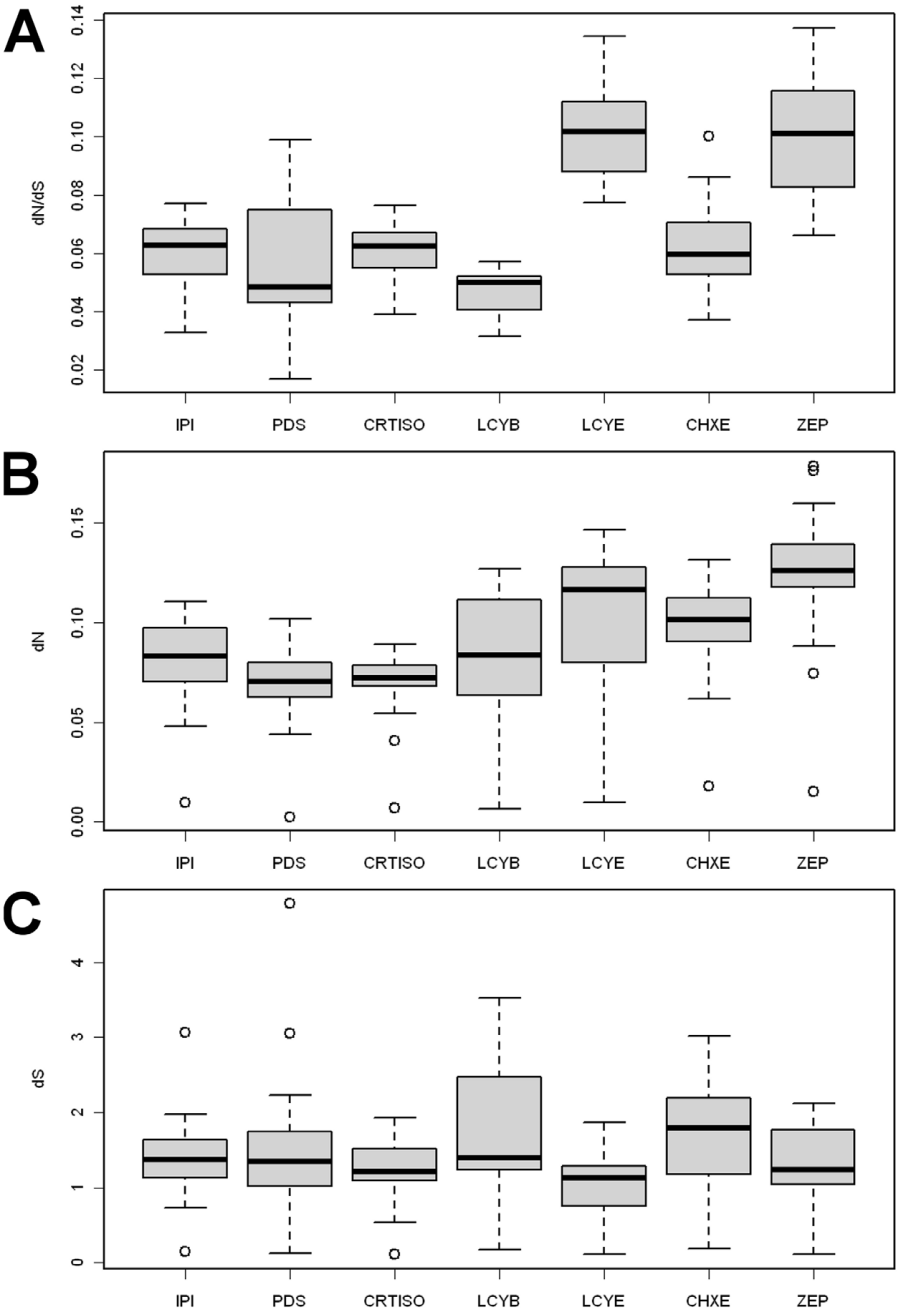
Role of nonsynonymous and synonymous substitution rates in *d_N_*/*d_S_* ratio variation. Distribution of (A) *d_N_*/*d_S_* ratio , (B) nonsynonymous substitution rate *d_N_* and (C) synonymous substitution rate *d_S_* calculated from pairwise comparison of seven dicots are displayed as a function of carotenoid biosynthesis genes. The genes are classified according to their pathway position.

The *d_N_*/*d_S_* ratio was positively correlated with pathway position (Kendall’s correlation test: *τ* = 0.26; *P* = 4×10^−5^; Figure 4A). In order to test the causes of *ω* variability between genes, i.e. mutation rate or purifying selection, correlation was tested for *d_N_* and *d_S_* separately. The *d_N_* was positively correlated with pathway position (*τ* = 0.33; *P* = 1×10^−7^; Figure 4B), whereas no correlation was found between *d_S_* and pathway position (*τ* = 0.05; *P* = 0.42; Figure 4C). In conclusion, the variations in the *ω* ratio observed between genes were closely linked with variations in the nonsynonymous substitution rate *d_N_* and positively correlated with pathway position.

**Figure 5:**
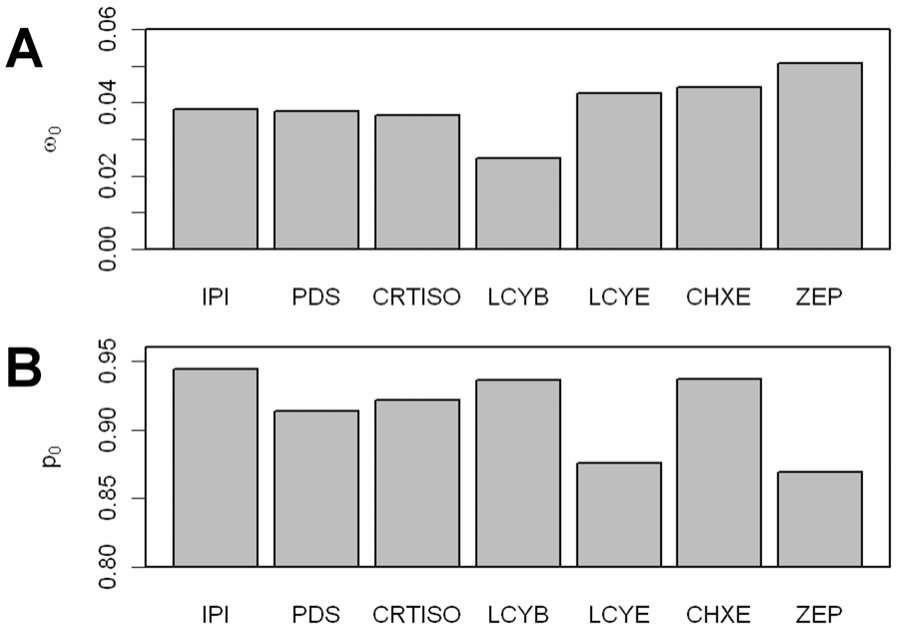
Constraint relaxation in the carotenoid biosynthesis pathway. (A) *ω_0_* and (B) ratio of codons with 0<*ω*_0_<1 (*p_0_*) are displayed as a function of carotenoid biosynthesis pathway genes. Values shown were estimated by the M1a model calculated by CODEML [65]. The genes are sorted according to their pathway position.

This result may be due to differences in the ratio of codons undergoing purifying selection and in the strength of purifying selection applied to these codons. For each gene, the M1a model, which expected some codons with 0<*ω_0_*<1 (purifying selection) and others with *ω_1_* = 1 (neutrality), significantly improved the likelihood in comparison with the M0 model which assumed all codons evolved neutrally (*P*<0.001; Table 1), indicating that some codons within carotenoid biosynthesis genes evolved under purifying selection. The proportion of codons that evolved under purifying selection (*p_0_*) was high, but varied from 87% in *ZEP* to 94% in *IPI* (Table 1; Figure 5B). The three genes *LCYE*, *CHXE* and *ZEP*, acting downstream in the pathway, showed the highest values of *ω_0_*, with 0.043, 0.044 and 0.051 respectively (Table 1; Figure 5A). The *ω_0_* values of the other genes were inferior to 0.039. This result confirmed that purifying selection is less important in downstream genes than upstream genes in carotenoid biosynthesis pathway.

### Selection Signatures at *PDS*, an Upstream Gene

Carrot domestication has led to a change between an uncolored root in wild carrots to a root with sometimes high carotenoid levels in cultivated carrots. This change should have been obtained by positive selection and should be linked to a reduction of diversity for targeted genes. In order to detect nucleotide diversity variations in carotenoid biosynthesis genes, we used HKA neutrality tests. Pairwise HKA tests gave significant results for all comparisons implicating *PDS*, i.e. pairwise comparisons between *PDS* and *IPI*, *CHXE* or *ZEP* (*P*<0.01) and between *PDS* and *CRTISO*, *LCYB1* or *LCYE* (*P*<0.001). The results of all other pairwise comparisons were not significant (data not shown). This result was confirmed by the ML-HKA test [25], for which a model allowing selection for *PDS* showed highly significant improvement to the likelihood compared with the neutral model (χ^2^ = 19.62, df = 1, *P*<0.001). The maximum likelihood estimate of the selection parameter *k* was 0.16, suggesting a six-fold decrease in diversity over neutral expectation at this locus, in comparison with other genes. *PDS* showed low nucleotide diversity (*π* = 0.003) in the carrot set but high sequence divergence between the carrot and the tuberous-rooted chervil, with 216 fixed differences between the two species for the 911 sites compared. These marked differences between species suggest a selective sweep or background selection in the carrot lineage, or a modification in local mutation rates around *PDS* after divergence of the two species. In order to test these hypotheses, pairwise HKA tests were then applied to coding regions only (153 sites). Among all comparisons, a single significant departure from neutrality was revealed in the *PDS*-*LCYE* pairwise comparison (*P*<0.05), suggesting that the specific ratio between polymorphism and divergence shown for *PDS* in both introns and exons was less convincing for exonic regions only. The main difference between the effect of background selection and selective sweep is that the latter results in a deviation toward an excess of low-frequency alleles, while the former does not [26]. Even if *PDS* showed the lowest Tajima’s *D* statistic (*D* = −0.622; *P*>0.05), it did not significantly deviate from the expectations under the divergence model, as may be expected with selective sweep. However, the Tajima’s test may fail to detect recent selective sweeps because of a lack of regeneration of polymorphism since the selection event. We thus cannot conclude on whether the reduced polymorphism at *PDS* was obtained by background selection or by a selective sweep during carrot domestication.

If *PDS* experienced positive selection in carrot, the phylogenetic analysis should reveal an accelerated evolutionary rate for *PDS* in carrot by comparison to other dicots. We analyzed the ratios of nonsynonymous (*d_N_*) to synonymous substitutions (*d_S_*) in protein coding regions within carotenoid biosynthesis genes in several dicots in order to detect positive selection (Table 1). Differences in *d_N_*/*d_S_* (*ω*) ratios among lineages were detected for *PDS, CHXE* (likelihood ratio test for the pair M0–M1; *P*<0.01) and for *ZEP* (*P*<0.001). The carrot lineage did not exhibit a significantly different *d_N_*/*d_S_* ratio from other background branches (likelihood ratio test for the pair M0–M2; *P*>0.05). However, the *PDS* gene gave a result close to the 5% threshold (*P* = 0.058). Carrot-lineage specific *ω_1_* = 0.0937 was higher than background lineages *ω_0_* =  0.0620. This tendency to an acceleration of the non-synonymous rate compared with the synonymous rate of substitution in the carrot is congruent with the low polymorphism/divergence ratio found for *PDS* in HKA tests and suggests positive selection at *PDS* in the carrot.

### Selection around the Metabolic Node Lycopene

In addition to pathway position, pathway reticulation can influence the evolution of metabolic pathway genes. Departures from expectations under the divergence model were tested for the seven carotenoid biosynthesis genes (Table 2; Table 3). Significant tests were only found for genes surrounding the lycopene pathway node: *CRTISO*, *LCYE* and *LCYB1*.

**Table 2:** Tajima’s *D* and normalized Fay and Wu’s *H* in the pooled sample and geographical groups.

	POOLED SAMPLE				WEST				EAST			
Gene	*D*	p^a^	*H*	p^b^	*D*	p^a^	*H*	p^b^	*D*	p^a^	*H*	p^b^
***IPI***	−0.11	0.809	−0.45	0.453	0.06	0.891	−0.39	0.516	0.02	0.810	−0.10	0.663
***PDS***	−0.62	0.420	−1.36	0.173	−0.58	0.425	−0.52	0.443	−0.12	0.690	−1.87	0.106
***CRTISO***	**2.64**	**0.016** *	−0.77	0.321	**3.12**	**0.001** **	0.06	0.748	−**2.51**	**0.000** ***	−**4.19**	**0.003** **
***LCYB1***	1.61	0.143	−0.93	0.273	−0.20	0.680	−2.21	0.076	1.86	0.110	−0.38	0.493
***LCYE***	**2.31**	**0.029** *	−1.05	0.251	**3.00**	**0.002** **	−0.22	0.585	**3.03**	**0.001** **	−0.26	0.563
***CHXE***	−0.12	0.809	−1.11	0.225	−1.83	0.022*	−1.92	0.106	1.76	0.134	−0.15	0.62
***ZEP***	0.76	0.517	−0.96	0.273	−1.03	0.182	−2.37	0.065	1.66	0.174	−1.07	0.261

a Probability of two-tailed test based on the rank of Tajima’s *D* for the candidate genes by comparison with the expected distribution obtained by approximate Bayesian computation simulations under the divergence model.

b Probability of one-tailed test based on the rank of normalized Fay and Wu’s *H* for the candidate genes by comparison with the expected distribution obtained by approximate Bayesian computation simulations under the divergence model.

* : *P*<0.05;

** : *P*<0.01;

*** : *P*<0.001.

**Table 3:** Comparison of *F_ST_* in the geographical and color groups.

		Geographical groups			Color groups		
Gene	*θ_w_*	*F_ST_*	*p* a	*n* b	*F_ST_*	*p* a	*n* b
***IPI***	7.5	−0.007	0.348	650	0.119	0.116	774
***PDS***	5.0	0.019	0.673	113	0.081	0.150	173
***CRTISO***	17.1	**0.336**	**0.008** **	1227	0.034	0.530	1166
***LCYB1***	4.5	0.072	0.658	313	**0.218**	**0.013** *	468
***LCYE***	11.3	−**0.044**	**0.000** ***	1525	0.154	0.088	1450
***CHXE***	9.5	0.103	0.387	1164	0.076	0.280	1330
***ZEP***	5.0	0.266	0.059	540	0.148	0.066	722

a Probability of two-tailed test based on the rank of *F_ST_* for the candidate genes by comparison with the expected distribution obtained by approximate Bayesian computation simulations under the divergence model.

* : *P*<0.05;

** : *P*<0.01;

*** : *P*<0.001.

b Number of simulations used to test the significance of *F_ST_*.

The *CRTISO* gene showed a significant positive Tajima’s *D* in the pooled sample (*D* = 2.64; *P*<0.05), showing an excess of intermediate-frequency polymorphisms in this group. Only the Western group showed a similar pattern (*D* = 3.12; *P*<0.01) in *CRTISO* while Tajima’s *D* was significantly negative in the Eastern group (*D* = −2.51; *P*<0.001), suggesting an excess of low-frequency polymorphisms in this group for *CRTISO*. A highly significant differentiation was found between Western and Eastern groups for *CRTISO* (*F_ST_* = 0.336; *P*<0.01). Interestingly, we found a significantly negative normalized Fay and Wu’s *H* in the Eastern group (*H* = −4.19; *P*<0.01), indicating an excess of high-frequency derivate polymorphisms and suggesting a selective sweep at *CRTISO* in the Eastern group.

The gene *LCYE* also showed a significant positive Tajima’s *D* in the pooled sample (*D* = 2.31; *P*<0.05). Contrary to *CRTISO*, this excess of intermediate frequency polymorphisms was independent of population structure, as significant positive Tajima’s *D* values were also found for this gene in both Western and Eastern groups (*D* = 3; *P*<0.01 and *D* = 3.03; *P*<0.01, respectively). This result was confirmed by a significantly low differentiation between Western and Eastern populations (*F_ST_* = −0.044; *P*<0.001). *LCYE* may have been subjected to balancing selection at the subspecies level, as population structure-independent balancing selection is expected to decrease population differentiation [27].

The gene *LCYB1* is the only one with a significant *F_ST_* for color groups (*F_ST_* = 0.218; *P*<0.05). This result suggests that the polymorphism at this gene is structured by root color and may be related to breeding for color diversification.

The excess of intermediate frequency polymorphisms in *CRTISO* and *LCYE* as well as the high differentiation of color groups for *LCYB1* make feel that these three genes surrounding the metabolic node lycopene may have experienced balancing selection in carrot. Balancing selection generally leads to an increase of diversity. HKA test was used to test for specific nucleotide diversity levels in these three genes. A model that assumes selection at these genes significantly improved the likelihood in comparison with the neutral model (ML-HKA test; χ^2^ = 12.39, df = 3, *P*<0.01). The maximum likelihood estimate of the selection parameter *k* for *CRTISO* (*k* = 2.62), *LCYB1* (*k* = 2.38) and *LCYE* (*k* = 2.88) suggests a twofold increase in diversity over neutral expectations at these loci, in comparison with the other carotenoid biosynthesis genes analyzed. The excess of variability and the deviation of allele frequency spectrum toward intermediate frequency suggest that *CRTISO*, *LCYE* and *LCYB1*, acting at the center of the carotenoid pathway and surrounding the metabolic node lycopene, may have been evolving non-neutrally in a pattern consistent with balancing selection.

## Discussion

### Major Selective Constraints on Upstream Genes Versus Relaxed Selective Constraints on Downstream Genes

The analysis of the *d_N_* /*d_S_* ratio revealed variations in the level of purifying selection in the pathway. Our results are consistent with a relaxed constraint on downstream carotenoid biosynthesis genes, in comparison with more upstream genes, and complement those of Livingstone and Anderson in the same pathway [14]. These authors showed that the downstream gene *ZEP* has more codons evolving under relaxed constraints than three more upstream genes *PDS*, *ZDS*, and *LCYB.* Similar conclusions were reached in studies of the *d_N_*/*d_S_* ratio in the anthocyanin [6] and terpenoid pathways [5]. However, the pathway position does not explain the particular evolutionary rate of *LCYB*. *LCYB* had the lowest *d_N_* /*d_S_* ratio, yet was located at the same level of the pathway as *LCYE* (Figure 3). LCYB was shown to act once in the lutein branch and twice in the β-carotene branch, while LCYE only acts in the lutein branch (Figure 1). Higher pleiotropy in the pathway for LCYB may have contributed to the high selective constraints observed for this gene.

A relationship between a differential selection pattern and gene position in a metabolic pathway has rarely been demonstrated by studying infraspecific polymorphism (but see [8] and [28]). Interestingly, we found that downstream carotenoid biosynthesis genes showed more commonly deviations from neutrality expectations in cultivated carrot than upstream genes, especially *IPI* and *PDS* (Figure 2). One possible explanation for this result is that upstream genes are more constrained than downstream genes. Results obtained for *d_N_*/*d_S_* comparisons reinforce this hypothesis (Figure 4). Moreover, *IPI* and *PDS* exhibited a singular “star-like” haplotype network, although haplotype networks structured with at least two haplogroups were found for other loci (data not shown). This result is consistent with constraints preventing haplotype differentiation in these two upstream genes. A second possible explanation is that downstream genes are more prone to positive or balancing selection than upstream genes. In the context of artificial selection in carrot, this pattern may be expected, as the major carotenoids that accumulate in carrot germplasm (β-carotene, α-carotene, lutein and lycopene) are products of central or downstream enzymes (Figure 1). More generally, among the seven carotenoid biosynthesis genes whose *d_N_*/*d_S_* ratio was analyzed by Ramsay et al. [5], the two genes showing evidence of positively selected codons were *LCYB* and *CHXE* which act downstream in the pathway. For the purpose of comparison, differential nonsynonymous substitution rates in anthocyanin genes in *Ipomea* were explained by relaxed constraints on the downstream genes rather than by positive selection in this pathway, as positive selection was not detected in this pathway [7], [8]. In the carotenoid biosynthesis pathway, we can suppose that the two processes influenced the nucleotide patterns.

Two factors have been proposed to explain the stronger evolutionary constraints on upstream than downstream genes: firstly, upstream enzymes exert greater control of metabolic fluxes than downstream enzymes, and secondly, upstream enzymes influence more end products than downstream enzymes [1]. However, weaker selective constraints on downstream genes than on upstream genes cannot be assumed to apply to all metabolic pathways. For example, no correlation was detected between constraints and the position of the gene in gibberellin pathway in plants [10], nor in starch pathway in rice [11]. These results suggest that the nature of selection in a metabolic pathway depends on the function of the pathway.

Linking the nature of selection and the function of the carotenoid pathway is challenging. Beyond coloring fruits, petals and some roots [29], carotenoids act as accessory pigments in photosynthesis and are involved in dissipating excess excitation energy of chlorophyll molecules as heat by non-photochemical quenching (NPQ), a fundamental process to preserve photosynthetic activity [30]. The dual role of carotenoids in the plant probably explains the duplication of some carotenoid biosynthesis genes and the subsequent specialization of the two homologous genes in fruits and flowers or in leaves in tomato [31]. In carrot, we do not know if the same genes control the occurrence of carotenoids in roots and leaves. Therefore, beyond the fact that they may have undergone human selection for colored roots, carotenoid pathway genes may have been targeted by a high purifying selection in order to maintain the required levels of carotenoids in leaves.

### Positive Selection of an Upstream Gene during Crop Domestication

Due to their role in controlling metabolic fluxes and to their epistatic role on following steps of metabolic pathways, upstream genes are probably strategic aims during crop domestication and breeding. As an example, positive selection at *Y1* encoding PSY, the first enzyme of the pathway, has led to the increase of yellow/orange endosperm phenotype in maize in the 20^th^ century [16]. Reduced expression levels of *PSY1* and *PSY2* and absence of PSY enzyme in wild and cultivated white carrots compared with orange carrots, suggest that *PSY1* and *PSY2* expression is the rate-limiting step for carotenoid accumulation in white carrots [32]. Neither *PSY1* nor *PSY2* co-located with QTLs for carotenoid occurrence in carrot [33], suggesting that the gene underlying the occurrence of carotenoids in carrot root may instead be another gene, maybe a transcription factor. A major QTL, *Y*, controlling accumulation of xanthophylls, mapped near *PDS*, a gene encoding the second enzyme of the carotenoid pathway [24], [33]. Our results suggest that *PDS* may have undergone positive selection in the carrot. Xanthophylls are major pigments in the root of yellow carrots [18]. It has been hypothesized that a mutation at the *Y* locus may have influenced the selection of cultivated yellow carrots from wild white carrots, during domestication [33]. The major reduction in diversity observed in *PDS* in cultivated carrots reinforces this hypothesis, as artificial selection during domestication is expected to lead to a greater decrease in diversity around selection targets than a bottleneck effect. Selection at *PDS* may have influenced metabolic fluxes allocated to the carotenoid pathway, as PDS acts early in this pathway (Figure 1). Further investigation is needed to confirm this reduction in diversity by studying wild progenitors or relatives, and to determine whether *PDS* was directly targeted by selection or underwent a selective sweep by selection at a linked gene.

### Balancing Selection for Genes Surrounding a Metabolic Node

In addition to the upstream, central or downstream position of genes in the metabolic pathway, the position of the genes with respect to metabolic nodes has been postulated to influence their selection patterns [1]. Our results in the cultivated carrot evidence particular signatures of selection in genes surrounding the lycopene, a metabolic node in the carotenoid biosynthesis pathway (Figure 1). The polymorphism patterns of the genes *CRTISO* and *LCYE* are consistent with balancing selection, while the differentiation analyses suggest that diversifying selection may have impacted *LCYB1* during carrot breeding for root color. Among the seven carotenoid biosynthesis genes investigated in the cultivated carrot, the highest silent-site nucleotide diversity was found for the three genes *CRTISO* (*π_sil_* = 0.0440), *LCYB1* (*π_sil_* = 0.0297) and *LCYE* (*π_sil_* = 0.0273) [34]. Large intragenic LD was found for *LCYE* (average *r^2^* = 0.93) and *CRTISO* (average *r^2^* = 0.86), while *LCYB1* (average *r^2^* = 0.52) showed intermediate LD [34]. These results are consistent with expectations under balancing selection, i.e. an increase in nucleotide diversity at closely linked neutral sites of the targeted site under balancing selection, and a high linkage disequilibrium [35].

Understanding the biological function of the maintenance of diversity at *CRTISO*, *LCYB1* and *LCYE* is challenging. These three genes surround lycopene in the carotenoid biosynthesis pathway (Figure 1). Lycopene is the direct precursor of carotenoids produced in both metabolic branches of this pathway. It thus represents a central metabolic node of the carotenoid biosynthesis pathway [36]. Metabolic flux could be oriented toward one branch or another by genes acting downstream from lycopene. Maintenance of the genetic variation of these genes in cultivated carrot due to differential metabolic flux allocation toward branches leading to lutein or β-carotene among color types may explain the excess of polymorphism and intermediate frequency alleles or the high differentiation between color groups shown at *CRTISO*, *LCYB1* and *LCYE*. Similarly, genes involved in channeling metabolic fluxes downstream from metabolic nodes were found to be the targets of adaptive selection in the central metabolism of *Drosophila* [3] and in the starch pathway in maize [4].

Although they are centrally located in the carotenoid biosynthesis pathway and surround the metabolic node lycopene, these three genes probably play unequal roles in controlling metabolic fluxes. As *CRTISO* is located directly upstream from lycopene, this gene may not influence flux allocation (Figure 1). *LCYB1* acts downstream from lycopene but both in metabolic branches leading to lutein and to β-carotene, suggesting that this gene may not be the most important gene influencing flux allocation. *LCYE* acts downstream from lycopene, only in the branch leading to lutein, and consequently may control metabolic fluxes in the carotenoid biosynthesis pathway after lycopene. In maize germplasm, variation at *LCYE* alters the flux down lutein versus β-carotene branches, confirming this gene as the main determinant of flux allocation between branches of this pathway [37]. Besides gene position in the pathway, the signal of balancing selection detected in carrot for *CRTISO*, *LCYB1* and above all for *LCYE* confirms that reticulation of the pathway is a further factor influencing differential selection in the pathway.

### Conclusion

A putative signature of selection during domestication of carrot was found at the upstream *PDS* gene, maybe in relation to the control of metabolic flux by upstream genes. Genes surrounding lycopene exhibited nucleotide patterns consistent with balancing selection in carrot, which suggests that genes near metabolic nodes are selection targets in metabolic pathways. Finally, this study showed a relaxation of evolutionary constraints along the carotenoid biosynthesis pathway, both in cultivated carrot and in dicots.

## Materials and Methods

### Carrot Sample

For population genetics analyses, we used a sample of 46 cultivars of carrot (*Daucus carota* L. ssp. *sativus*), each one represented by a single individual [34] (Table S1). This sample was subdivided into three sets for neutrality tests: (i) sub-species, hereafter “pooled sample”, i.e. 46 individuals, (ii) geographical groups, i.e. Western and Eastern groups, defined according a genetic structure analysis using 17 microsatellite loci [34], and (iii) color groups, i.e. individuals with white, yellow, orange, red or purple roots. A wild individual of tuberous-rooted chervil (*Chaerophyllum bulbosum* L.), a related *Apiaceae*, was used for analyses requiring an outgroup.

### Sequence Dataset for Carrots

Seven carotenoid biosynthesis genes were used: *IPI*, *PDS*, *CRTISO*, *LCYB1*, *LCYE*, *CHXE* and *ZEP* (Figure 1). We chose genes distributed along the pathway, preferentially known to be single copy genes [24], except *LCYB1*, or according to their implication in color determinism in other species. Amplified regions contained both introns and exons. PCR, cloning and sequencing conditions, and primers used to amplify these sequences are described in [34]. Three anonymous loci, *B1D*, *JW3D*, *SB4A*, were generated from random amplified polymorphic DNA fragments. In the search for sequence identity with published nucleotide sequences using TBLASTX [38], these loci were chosen for their low scores. The primers used were 5′-ttctctttgggtcaagtggattca-3′ (Forward) and 5′-tcgctcctgccatatcacataca-3′ (Reverse) for *B1D*; 5′-ggctagagtggaggcgtgaa-3′ (Forward) and 5′-gctcactgaaggatttgatttgaa-3′ (Reverse) for *JW3D*; 5′-agcgcattgaaatggaggtttt-3′ (Forward) and 5′-aggctagcattgctctcttgatca-3′ (Reverse) for *SB4A*. The same PCR conditions as in [34] were used, with an annealing temperature of 54°C for *B1D* and *JW3D*, and of 55°C for *SB4A*. These three anonymous DNA sequences, and 17 microsatellite loci already genotyped for this sample [34] were used as control loci to model the demographic history of the sample. All the sequences were deposited as GenBank accessions JX100840-JX101319.

### Sequence Polymorphism

DNA sequences were computed using DnaSP 4.9 [39]. Sites with alignment gaps were excluded from analyses. Nucleotide polymorphism *θ_w_* [40], and nucleotide diversity *π* [41] for silent sites (i.e., intronic regions plus synonymous sites) were calculated for each locus.

### Demographic Modeling

One major drawback of signatures of selection is the confounding effect of demographic events and selection. For example, an excess of intermediate frequency variation is consistent with balancing selection but may also be driven by population scale events like population subdivision [42]. Therefore, the genetic differentiation between Western and Eastern cultivated carrots must be taken into account when testing carotenoid biosynthesis genes for selection [34]. Using control loci, demographic models that are more realistic than the standard neutral model (SNM) can be designed to identify candidate genes straying from expectations [43], [44].

To determine the impact of the population structure of the sample [34] on neutrality tests, the demographic history of the sample was simulated using approximate Bayesian computation [45]. The model, hereafter called ‘divergence model’, included two populations corresponding to the Western and the Eastern populations described in [34], assuming constant effective population sizes, *N_W_* and *N_E_* respectively. At *T_d_* generations in the past, these two populations diverged from an ancestral population of an effective population size *N_A_*. Following this model, datasets including 17 autosomal diploid microsatellites and three autosomal haploid DNA sequences were simulated. Microsatellite loci were simulated using the generalized stepwise mutation model with the mean mutation rate *µ_SSR_* and the parameter of the geometric distribution *P_SSR_*. The same motif size and allele range as in observed data were used for the simulations. DNA sequences were simulated using the Jukes-Cantor model [46] with the mean mutation rate *µ_seq_*. Prior distribution of parameters is described in Table S2. According to the spread of the cultivated carrot to Europe via the Middle East and North Africa, between the 10^th^ and the 12^th^ centuries [19] and of biennial reproduction of carrot, priors for *T_d_* follow a normal distribution such as *X ∼* N(500,100) truncated such that 350 ≤ *X* ≤ 750. A total of 10^6^ approximate Bayesian computation simulations were released by DIYABC v.1.0 software [47]. Summary statistics were chosen for their correlation with one or several parameters to be estimated (Table S3). Summary statistics retained for the analysis are the mean number of alleles across loci in each population, *F_ST_* between the two populations [48], and the shared allele distance between each population [49] for microsatellite loci; the number of distinct haplotypes in each population and in the pooled sample, the number of segregating sites in the pooled sample and *F_ST_* between the two populations [50] for DNA sequences. Posterior distributions of parameters were estimated through a local linear regression procedure [45], with a threshold of 10^−2^ (Figure S1). The model was checked by comparing the distribution of summary statistics for priors, predictive posteriors and observed datasets in a principal component analysis (PCA) [47] (Figure S2). The fit of the model-posterior combination to the observed data was tested by the rank of summary statistics for the observed dataset in the distribution of the same summary statistics obtained from the posterior predictive distribution [47] (Table S4). The description and the checking of the divergence model used to take the population subdivision of carrot [34] into account in neutrality tests are in Text S1.

### Coalescence-based Neutrality Tests

Tajima’s *D* [51], normalized Fay and Wu’s *H* [52] and *F_ST_* [50] were calculated using polymorphic sites of the seven carotenoid biosynthesis candidate genes. Parameter posteriors estimated by approximate Bayesian computation analysis were used to test the significance of each statistic. Random combinations of effective population sizes *N_W_*, *N_E_*, *N_A_*, divergence time *T_D_* and mean DNA sequence mutation rate *µ_seq_* were resampled in the posterior distribution using the algorithm described in [53]. These parameter combinations were used to simulate datasets following the same demographic model as for approximate Bayesian computation evaluation, using msABC [54]. A set of 10^4^ simulations was run for each of the seven carotenoid biosynthesis genes, taking the length of each sequence fragment into account. For the seven candidate genes, we estimated the rate of misorientations when determining ancestral states in carrot polymorphism data by comparison with the outgroup *Chaerophyllum bulbosum* L. [55]. We generated simulated datasets using the divergence model with a similar back mutation rate, as ignoring misorientations could influence neutrality tests based on Fay and Wu’s *H* [44]. For each of the seven carotenoid biosynthesis genes, the rank of observed Tajima’s *D* and normalized Fay and Wu’s *H* in their respective expected distribution were calculated according to the divergence model (Figure S3). For the pooled sample and the Western and the Eastern samples, Tajima’s *D*, normalized Fay and Wu’s *H* and *F_ST_* were directly calculated for simulations using msABC [54]. Simulated sequence datasets for color groups were obtained by sampling as many sequences from the Western and the Eastern populations as observed in each color group. Neutrality statistics were then calculated using SEQLIB (seqlib.sourceforge.net). The rank value was used to make a two-tailed test for Tajima’s *D* and a one-tailed test for lowest normalized Fay and Wu’s *H* values. As *F_ST_* is influenced by the mutation rate [56], the rank of *F_ST_* observed for a given gene was calculated by comparison with the expected distribution of *F_ST_* in simulated datasets sharing similar *θ_w_* per gene ± 1.5 (Figure S4). The rank value obtained for *F_ST_* was used to make a two-tailed test. Prior and posterior parameter distributions, and neutrality statistics distributions for carotenoid biosynthesis genes relative to simulated datasets were plotted using R software [57]. The description of the neutral expectations under the divergence model is in Text S1. Hudson-Kreitman-Aguadé (HKA) tests, based on comparisons of divergence and variability between loci, were computed using DnaSP [58]. A maximum-likelihood extension of the HKA test was used [25]. For each locus, the DNA sequence of tuberous-rooted chervil was used as outgroup to carry out HKA and Fay and Wu’s *H* tests.

### Relationship of Neutrality Test Statistics and Pathway Position in the Carrot Dataset

The p-values obtained for neutrality tests based on Tajima’s *D*, Fay and Wu’s *H* and *F_ST_* in the pooled sample, geographical groups and color groups were pooled for each gene. The Kendall’s rank correlation coefficient *τ* was calculated by comparing p-values for neutrality statistics, and pathway position. Pathway position was established relative to the most upstream gene (*IPI)* and corresponds to the number of different enzymes involved between *IPI* and the gene considered. If a gene, e.g. *LCYB1*, *LCYE* and *CHXE*, was involved at different metabolic steps in the carotenoid pathway, to calculate its position in the pathway, we considered the most upstream step. Pathway position indexes for each of the seven genes are shown in Figure 1.

### Sequence Dataset for the Phylogenetic Analysis

To screen for selection pressures along coding regions of carotenoid biosynthesis genes and to evaluate selective constraints on nucleotide substitutions, we calculated the ratio of nonsynonymous (*d_N_*) and synonymous substitutions (*d_S_*) in protein coding regions within carotenoid biosynthesis genes in several species [59], [60]. We used the coding sequence of the seven carotenoid biosynthesis genes found in the dark orange carrot cultivar ‘B493’ [24] to search for orthologous DNA sequences using TBLASTN [38] against all plant gene indices in GenBank sequence database. The database was consulted on June 11, 2011. Complete orthologous sequences of the seven carotenoid genes were retrieved for *Solanum lycopersicum* L., *Vitis vinifera* L., *Populus trichocarpa* Torr. & A.Gray, *Ricinus communis* L., *Arabidopsis thaliana* (L.) Heynh. and *Arabidopsis lyrata* (L.) O’Kane & Al-Shehbaz (Table S5). When several copies of an ortholog were found in one species, we chose the one with the highest BLAST E-value. Sequences were trimmed down to the coding sequences and then translated using BioEdit 7.0.5.3 [61]. Peptide sequence alignments were created using ClustalW [62]. The occurrence of chloroplast leader sequences was predicted using the ChloroP 1.1 Server [63]. The DNA sequences corresponding to chloroplast leader sequences were removed and alignments were then adjusted manually.

### Analysis of Evolutionary Constraints

An unrooted phylogenetic tree was built for each gene, based on the neighbor joining method and the Jukes-Cantor nucleotide model using MEGA 5.05 software [64]. We used the CODEML program of the PAML program package to analyze several codon substitution models [65]. The models differed for parameter *ω* = *d_N_*/*d_S_*. Codons with *ω* = 1 are assumed to evolve neutrally, while codons with 0<*ω*<1 are assumed to evolve under purifying selection and codons with *ω*>1 are assumed to evolve under positive selection. The null model M0 assumes *ω* to be constant for all codons of the sequences analyzed and for all the branches concerned. We compared the likelihood of the null model M0 with two ‘branch models’ M1 and M2. M1 is the free ratios model which assumes an independent *ω* ratio for each branch. Model M2 assumes there are two *ω* ratios, one for the carrot branch and one for the rest of the tree, indicating selection in the carrot branch. We also used two ‘site models’ M1a (*Nearly Neutral*) and M2a (*Positive Selection*), allowing the *ω* ratio to vary among sites. M1a assumes that the sequence analyzed displays some codons with 0<*ω*<1 and other codons with *ω* = 1. M2a assumes that the sequence analyzed displays three classes of codons with 0<*ω*<1, *ω* = 1 and *ω*>1. The fit of the null model M0 versus a branch or a site model was evaluated by the likelihood ratio test. To check if carotenoid biosynthesis genes evolved under differential selective constraints, we tested the significance of differences in *ω* by comparing the likelihood obtained with the model M0 with the same model but constraining *ω*, as described in [7]. Two genes with *ω_1_* and *ω_2_* respectively have overlapped confidence intervals if there is no given *ω_f_* such as *ω_1_<ω_f_<ω_2_*, giving a higher likelihood than *ω_1_* or *ω_2_*. In the opposite case, the confidence intervals of *ω_1_* and *ω_2_* do not overlap, and consequently the two compared genes have statistically different *ω* values.

## Supporting Information

Figure S1 **Prior (dashed line) and posterior (solid line) distribution of approximate Bayesian computation model parameters.** Population sizes for Western group (*N_W_*), Eastern group (*N_E_*) and ancestral population (*N_A_*) are expressed as the absolute number of individuals and are assumed to be constant. Divergence time (*T_d_*) between Western and Eastern groups is expressed as the number of generations since divergence. Mean mutation rate for microsatellites *µ_seq_* is expressed as the number of mutations per site per generation. *P_SSR_* is the parameter of the geometric distribution in a generalized stepwise mutation model for microsatellites. Mean mutation rate *µ_seq_* for sequences is expressed as the number of substitutions per site per generation.
(TIF)

Figure S2 **Model checking.** Principal Component Analysis in the space of summary statistics was done for the observed dataset, prior distributions of parameters, and posterior predictive distribution of parameters. Only 105 points were plotted for prior distributions.
(TIF)

Figure S3 **Distribution of Tajima’s** ***D*** **and normalized Fay and Wu’s** ***H*** **simulated from posterior model parameters for pooled sample and geographical groups.** Dashed lines delineate the 95% confidence interval. Observed values for the seven carotenoid biosynthesis genes are shown. I: *IPI*; P: *PDS*; C: *CRTISO*; B: *LCYB1*; E: *LCYE*; X: *CHXE*; Z: *ZEP*; *y*-axis: distribution density.
(TIF)

Figure S4 **Distribution of** ***F_ST_*** **and** ***θ_w_*** **under the divergence model for comparison between Western and Eastern groups.** Observed values for the seven carotenoid biosynthesis genes are shown (filled circles). I: *IPI*; P: *PDS*; C: *CRTISO*; B: *LCYB1*; E: *LCYE*; X: *CHXE*; Z: *ZEP*.
(TIF)

Table S1 **Set of carrot cultivar samples used for population genetics analyses.**
(DOC)

Table S2 **Prior distributions of parameter values with the divergence model used during the approximate Bayesian computation analysis.**
(DOC)

Table S3 **Pearson correlation coefficients** ***r*** **between summary statistics and model parameters.**
(DOC)

Table S4 **Model checking by comparison of observed dataset and posterior predictive distribution.**
(DOC)

Table S5 **Accession number of genes used for the phylogenetic analysis.**
(DOC)

Text S1 **Construction and validation of the divergence model, and neutral expectations.**
(DOCX)
