# Correction: Differential Selection on Carotenoid Biosynthesis Genes as a Function of Gene Position in the Metabolic Pathway: A Study on the Carrot and Dicots

**DOI:** 10.1371/annotation/dcf13fd3-0489-4292-8a6b-d9f1146b5401

**Published:** 2012-10-22

**Authors:** Jérémy Clotault, Didier Peltier, Vanessa Soufflet-Freslon, Mathilde Briard, Emmanuel Geoffriau

There was an error in affiliation 1 for all listed authors. Affiliation 1 should be: Université d'Angers, UMR1345 Institut de Recherche en Horticulture et Semences, SFR 4207 QUASAV, PRES L'UNAM, Angers, France 

