# Supplementary figures and images for: Differential Selection on Carotenoid Biosynthesis Genes as a Function of Gene Position in the Metabolic Pathway: A Study on the Carrot and Dicots
Source: PLoS One. 2012 Jun 18;7(6):e38724. doi: 10.1371/journal.pone.0038724 (PMC3377682; doi:10.1371/journal.pone.0038724)

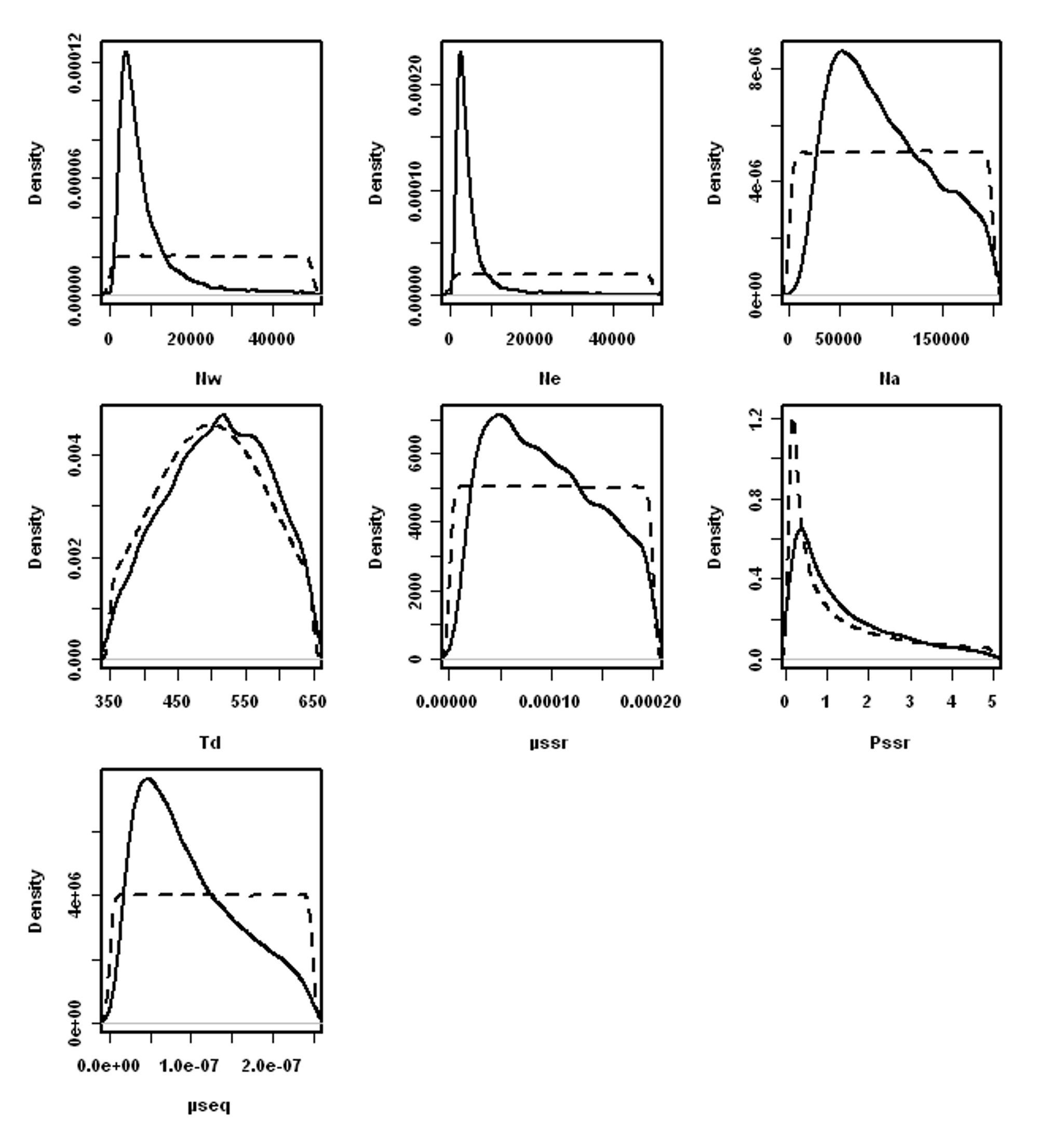

Supplement: Figure S1 — Prior (dashed line) and posterior (solid line) distribution of approximate Bayesian computation model parameters. Population sizes for Western group (NW), Eastern group (NE) and ancestral population (NA) are expressed as the absolute number of individuals and are assumed to be constant. Divergence time (Td) between Western and Eastern groups is expressed as the number of generations since divergence. Mean mutation rate for microsatellites µseq is expressed as the number of mutations per site per generation. PSSR is the parameter of the geometric distribution in a generalized stepwise mutation model for microsatellites. Mean mutation rate µseq for sequences is expressed as the number of substitutions per site per generation. (TIF) [file pone.0038724.s001.tif]

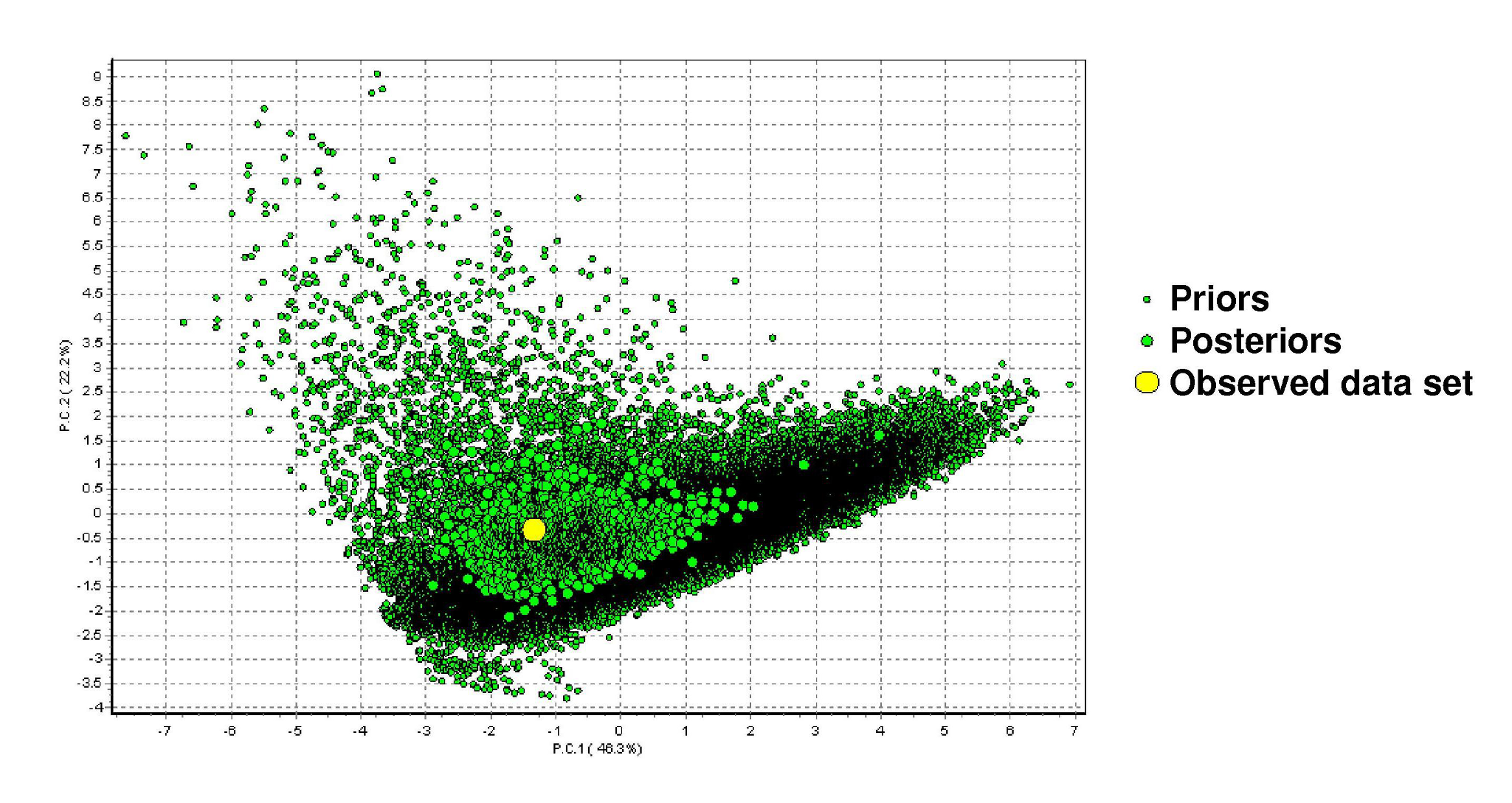

Supplement: Figure S2 — Model checking. Principal Component Analysis in the space of summary statistics was done for the observed dataset, prior distributions of parameters, and posterior predictive distribution of parameters. Only 105 points were plotted for prior distributions. (TIF) [file pone.0038724.s002.tif]

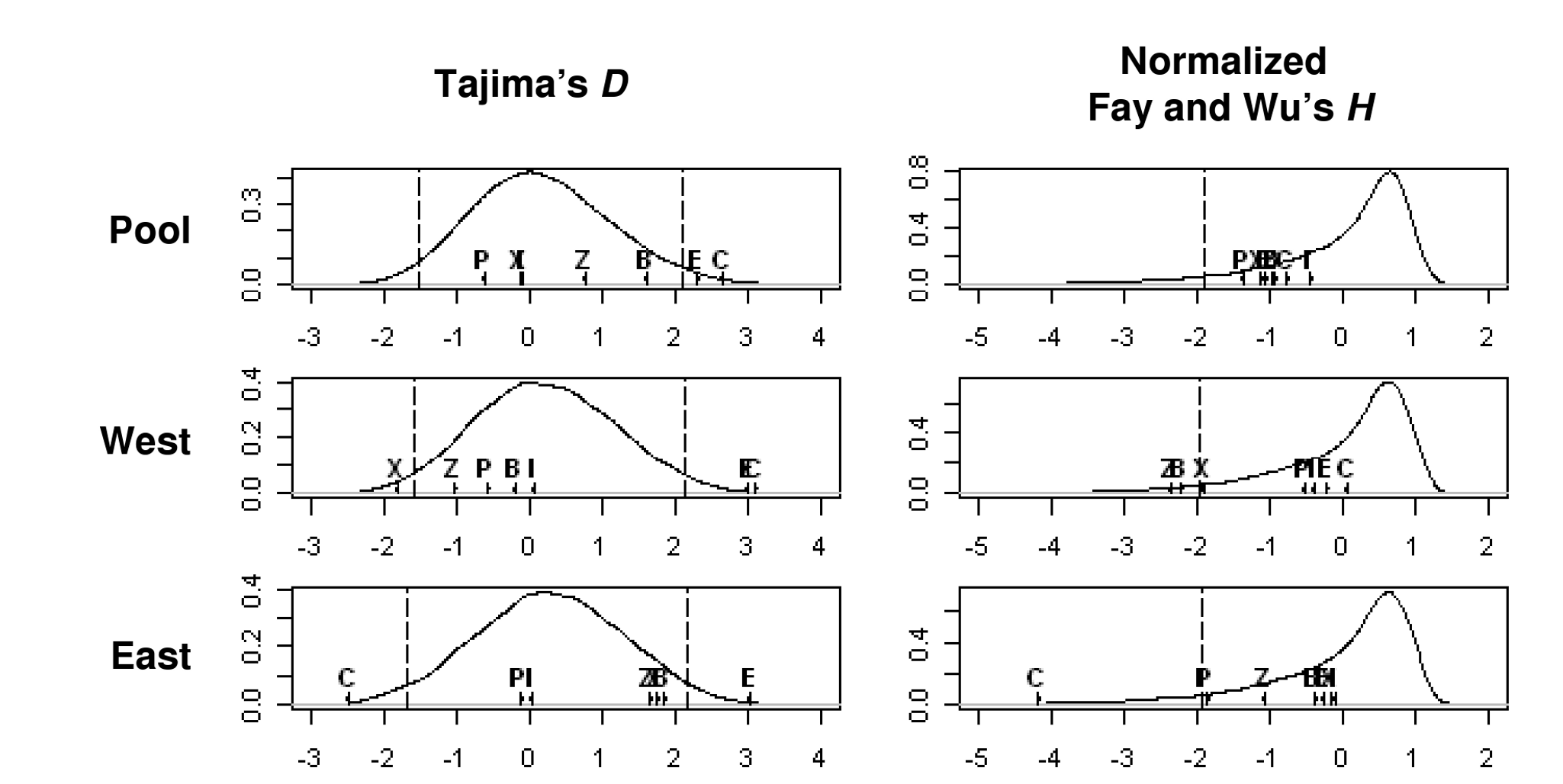

Supplement: Figure S3 — Distribution of Tajima’s D and normalized Fay and Wu’s H simulated from posterior model parameters for pooled sample and geographical groups. Dashed lines delineate the 95% confidence interval. Observed values for the seven carotenoid biosynthesis genes are shown. I: IPI; P: PDS; C: CRTISO; B: LCYB1; E: LCYE; X: CHXE; Z: ZEP; y-axis: distribution density. (TIF) [file pone.0038724.s003.tif]

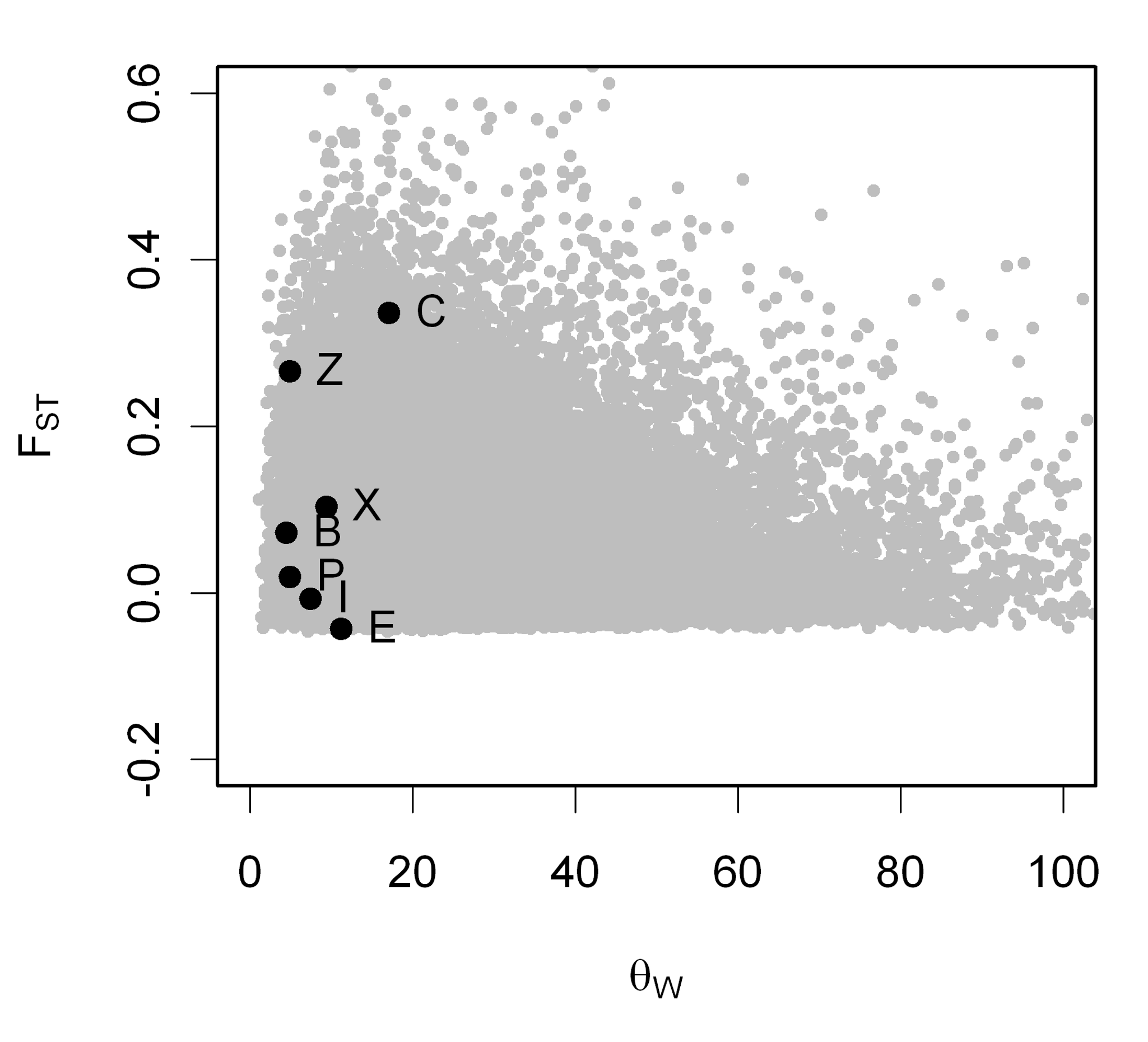

Supplement: Figure S4 — Distribution of FST and θw under the divergence model for comparison between Western and Eastern groups. Observed values for the seven carotenoid biosynthesis genes are shown (filled circles). I: IPI; P: PDS; C: CRTISO; B: LCYB1; E: LCYE; X: CHXE; Z: ZEP. (TIF) [file pone.0038724.s004.tif]
