# Supplementary material for: Differential Selection on Carotenoid Biosynthesis Genes as a Function of Gene Position in the Metabolic Pathway: A Study on the Carrot and Dicots
Source: PLoS One. 2012 Jun 18;7(6):e38724. doi: 10.1371/journal.pone.0038724 (PMC3377682; doi:10.1371/journal.pone.0038724)
Supplement: Table S1 — Set of carrot cultivar samples used for population genetics analyses. (DOC) [file pone.0038724.s005.doc]

Table S1. Set of carrot cultivar samples used for population genetics analyses

| **Code** | **Cultivar name** | **Geographical origin** | **Source** | **Geographical group^a^** | **Color group** |
| --- | --- | --- | --- | --- | --- |
| 106 | Snow White | USA | Territorial Seeds | Western | White |
| 111 | Amber White | USA | Kitchen Garden Seeds | Western | White |
| 301 | Sugarsnax | USA | Nunhems | Western | Orange |
| 104 | Blanche Collet Vert très Hors Terre | France | INH | Western | White |
| 105 | Blanche Demi Longue des Vosges | France | INH | Western | White |
| 210 | Jaune du Doubs | France | INH | Western | Yellow |
| 311 | Nantaise améliorée 4 | France | INH | Western | Orange |
| 312 | Bellot | France | INH | Western^b^ | Orange |
| 316 | Parisienne Market 2 | France | INH | Western | Orange |
| 337 | De Colmar à Cœur Rouge 2 | France | INH | Western | Orange |
| 109 | White Belgian | United Kingdom | HRI | Western | White |
| 100 | Kuttiger | Switzerland | INH | Western | White |
| 107 | White Satin | Holland | Bejo | Western | White |
| 201 | Yellowstone | Holland | Bejo | Western | Yellow |
| 313 | Amsterdam 2 Sweetheart | Holland | HRI | Western | Orange |
| 108 | Long White Green Top | Denmark | HRI | Western | White |
| 208 | Gelbe Lobbereicher | Germany | HRI | Western | Yellow |
| 303 | Boléro | Western Europe | Vilmorin | Western | Orange |
| 336 | Nandor | Western Europe | Clause Vegetable Seeds | Western | Orange |
| 339 | Premia | Western Europe | Syngenta Seeds | Western | Orange |
| 345 | Ceres | Western Europe | Clause Vegetable Seeds | Western | Orange |
| 348 | Adour | Western Europe | Syngenta Seeds | Western | Orange |
| 349 | Siroco | Western Europe | Vilmorin | Western | Orange |
| 928 | Nairobi | Western Europe | Bejo | Western | Orange |
| 901 | Tavola | Western Europe | Nunhems | Western | Orange |
| 526 | PD526 | Middle East | INH | Eastern | Purple |
| 520 | PM520 | Middle East | INH | Eastern | Purple |
| 521 | PD521 | Middle East | INH | Eastern | Purple |
| 522 | PA522 | Middle East | INH | Eastern | Purple |
| 366 | Mestnaya Zheltaya | Uzbekistan | VIR | Eastern | Orange |
| 227 | LR Mestnaya | Afghanistan | VIR | Eastern | Yellow |
| 514 | Afghan Purple | Afghanistan | HRI | Eastern | Purple |
| 403 | Annual Red Rawalpindi | Pakistan | HRI | Eastern | Red |
| 420 | Pusa Kesar | India | HRI | Eastern | Red |
| 516 | PD516 | Asia | INH | Eastern | Purple |
| 411 | Red Queen | India | Sungro | Eastern | Red |
| 224 | Ch-Wy | Asia | Mikado-Kyowa Seeds | Eastern | Yellow |
| 226 | YC226 | Asia | INH | Eastern | Yellow |
| 407 | Pink Selection | China | HRI | Eastern | Red |
| 421 | JF421 | Asia | INH | Eastern | Red |
| 307 | Kuroda | Japan | INH | Western | Orange |
| 426 | Honbeni Kintoki | Japan | Takii | Eastern | Red |
| 225 | Yellow BM | Asia | Vilmorin | Western | Yellow |
| 423 | Red Pink BM | Asia | Vilmorin | Eastern | Red |
| 400 | Nutrired | Asia / USA | Seminis | Eastern | Red |
| 200 | Kinby | Asia / USA | Johnny Seeds | Eastern | Yellow |

This set is identical to the set used in Clotault et al. (2010), without two admixed samples 515 and 500.

^a^Geographical groups represent assignation of samples obtained by analysis with 17 microsatellites using the model-based program STRUCTURE (Clotault et al. 2010).

^b^Not determined using STRUCTURE due to missing data but classified in ‘Western group’ as Bellot is a typical French cultivar and following assignation based on haplotypes of carotenoid biosynthesis genes (Clotault et al. 2010).
