# Supplementary material for: Differential Selection on Carotenoid Biosynthesis Genes as a Function of Gene Position in the Metabolic Pathway: A Study on the Carrot and Dicots
Source: PLoS One. 2012 Jun 18;7(6):e38724. doi: 10.1371/journal.pone.0038724 (PMC3377682; doi:10.1371/journal.pone.0038724)
Supplement: Table S2 — Prior distributions of parameter values with the divergence model used during the approximate Bayesian computation analysis. (DOC) [file pone.0038724.s006.doc]

Table S2. **Prior distributions of parameter values with the divergence model used during the approximate Bayesian computation analysis**

|  | **Distribution** | **Lower bound** | **Upper bound** | **Mean** | **Standard deviation** |
| --- | --- | --- | --- | --- | --- |
| ***N_W_*** | Uniform | 50 | 50000 |  |  |
| ***N_E_*** | Uniform | 50 | 50000 |  |  |
| ***T_d_*** | Normal | 350 | 650 | 500 | 100 |
| ***N_A_*** | Uniform | 1000 | 200000 |  |  |
| ***μ_SSR_*** | Uniform | 1×10^-6^ | 2×10^-4^ |  |  |
| ***P_SSR_*** | Log-uniform | 0.1 | 5 |  |  |
| ***μ_seq_*** | Uniform | 1×10^-9^ | 2.5×10^-7^ |  |  |

Population sizes in the Western group (*N_W_*), Eastern group (*N_E_*) and ancestral population (*N_A_*) are expressed as the absolute number of individuals and are assumed to be constant. Divergence time (*T_d_*) between Western and Eastern groups is expressed as the number of generations since divergence. Mean mutation rate for microsatellites *μ_seq_* is expressed as the number of mutations per site per generation. *P_SSR_* is the parameter of the geometric distribution in a generalized stepwise mutation model for microsatellites. Mean mutation rate *μ_seq_* for sequences is expressed as the number of substitutions per site per generation.
