# Supplementary material for: Differential Selection on Carotenoid Biosynthesis Genes as a Function of Gene Position in the Metabolic Pathway: A Study on the Carrot and Dicots
Source: PLoS One. 2012 Jun 18;7(6):e38724. doi: 10.1371/journal.pone.0038724 (PMC3377682; doi:10.1371/journal.pone.0038724)
Supplement: Table S3 — Pearson correlation coefficients r between summary statistics and model parameters. (DOC) [file pone.0038724.s007.doc]

Table S3. Pearson correlation coefficients *r* between summary statistics and model parameters

| **Loci** | **Summary statistics** | **Nw** | **Ne** | **Td** | **Na** | **μssr** | **Pssr** | **μseq** |
| --- | --- | --- | --- | --- | --- | --- | --- | --- |
| Microsatellites | Mean number of alleles across loci | 0.49 | 0.00 | -0.17 | 0.25 | 0.50 | -0.41 | 0.00 |
| Microsatellites | Mean number of alleles across loci | 0.00 | 0.49 | -0.16 | 0.25 | 0.50 | -0.41 | 0.00 |
| Microsatellites | Mean gene diversity across loci (Nei, 1987) | 0.42 | 0.00 | -0.13 | 0.25 | 0.42 | -0.44 | 0.00 |
| Microsatellites | Mean gene diversity across loci (Nei, 1987) | 0.00 | 0.41 | -0.13 | 0.25 | 0.43 | -0.44 | 0.00 |
| Microsatellites | Mean allele size variance across loci | 0.23 | 0.00 | -0.10 | 0.32 | 0.38 | -0.14 | 0.01 |
| Microsatellites | Mean allele size variance across loci | -0.01 | 0.22 | -0.09 | 0.32 | 0.39 | -0.14 | 0.00 |
| Microsatellites | Mean M index across loci (Garza and Williamson, 2001; Excoffier et al., 2005) | 0.10 | 0.01 | -0.03 | -0.15 | -0.03 | -0.11 | 0.00 |
| Microsatellites | Mean M index across loci (Garza and Williamson, 2001; Excoffier et al., 2005) | 0.01 | 0.06 | -0.02 | -0.17 | -0.07 | -0.08 | 0.01 |
| Microsatellites | *F_ST_* between two samples (Weir and Cockerham, 1984) | -0.45 | -0.40 | 0.38 | 0.03 | -0.13 | 0.09 | 0.00 |
| Microsatellites | Mean index of classification (1&2) (Rannala and Moutain, 1997; Pascual et al., 2007) | 0.03 | -0.32 | 0.24 | 0.37 | 0.47 | -0.45 | 0.00 |
| Microsatellites | Mean index of classification (1&2) (Rannala and Moutain, 1997; Pascual et al., 2007) | -0.36 | 0.02 | 0.26 | 0.36 | 0.45 | -0.43 | 0.00 |
| Microsatellites | (δμ )^2^ distance between two samples (Golstein et al., 1995) | -0.28 | -0.26 | 0.28 | 0.19 | 0.19 | -0.08 | 0.00 |
| Microsatellites | Mean number of alleles across loci | 0.21 | 0.17 | -0.08 | 0.32 | 0.61 | -0.47 | 0.00 |
| Microsatellites | Mean gene diversity across loci | 0.16 | 0.09 | -0.05 | 0.33 | 0.50 | -0.54 | 0.00 |
| Microsatellites | Mean allele size variance across loci | 0.07 | 0.04 | -0.03 | 0.36 | 0.42 | -0.15 | 0.00 |
| Microsatellites | Shared allele distance between two samples (Chakraborty and Jin, 1993) | 0.00 | 0.00 | -0.06 | -0.36 | -0.50 | 0.55 | 0.00 |
| DNA sequences | Number of distinct haplotypes | 0.56 | 0.00 | -0.18 | 0.18 | 0.00 | 0.00 | 0.53 |
| DNA sequences | Number of distinct haplotypes | 0.00 | 0.56 | -0.17 | 0.19 | 0.00 | 0.00 | 0.53 |
| DNA sequences | Number of segregating sites | 0.28 | 0.00 | -0.14 | 0.44 | -0.01 | -0.01 | 0.51 |
| DNA sequences | Number of segregating sites | 0.00 | 0.27 | -0.14 | 0.45 | -0.01 | 0.00 | 0.51 |
| DNA sequences | Mean pairwise difference | 0.23 | 0.00 | -0.12 | 0.45 | -0.01 | -0.01 | 0.48 |
| DNA sequences | Mean pairwise difference | 0.00 | 0.23 | -0.12 | 0.45 | -0.01 | -0.01 | 0.48 |
| DNA sequences | Variance of the number of pairwise differences | 0.09 | 0.00 | -0.04 | 0.34 | -0.01 | -0.01 | 0.34 |
| DNA sequences | Variance of the number of pairwise differences | 0.00 | 0.09 | -0.04 | 0.34 | -0.01 | -0.01 | 0.34 |
| DNA sequences | Tajima's D statistics (Tajima, 1989) | -0.13 | 0.00 | 0.03 | 0.37 | 0.00 | -0.01 | 0.08 |
| DNA sequences | Tajima's D statistics (Tajima, 1989) | 0.00 | -0.11 | 0.02 | 0.32 | 0.00 | -0.01 | 0.07 |
| DNA sequences | Number of private segregating sites | 0.29 | -0.26 | 0.08 | 0.31 | -0.01 | -0.01 | 0.44 |
| DNA sequences | Number of private segregating sites | -0.29 | 0.27 | 0.12 | 0.29 | -0.01 | 0.00 | 0.41 |
| DNA sequences | Mean of the numbers of the rarest nucleotide at segregating sites | 0.06 | 0.00 | 0.00 | 0.28 | -0.01 | 0.00 | 0.15 |
| DNA sequences | Mean of the numbers of the rarest nucleotide at segregating sites | 0.00 | 0.10 | -0.02 | 0.26 | 0.01 | 0.00 | 0.17 |
| DNA sequences | Variance of the numbers of the rarest nucleotide at segregating sites | 0.37 | 0.00 | -0.11 | 0.11 | 0.00 | 0.00 | 0.27 |
| DNA sequences | Variance of the numbers of the rarest nucleotide at segregating sites | 0.00 | 0.38 | -0.12 | 0.12 | 0.00 | 0.00 | 0.28 |
| DNA sequences | Number of distinct haplotypes in the pooled sample | 0.33 | 0.24 | -0.08 | 0.23 | 0.00 | 0.00 | 0.66 |
| DNA sequences | Number of segregating sites in the pooled sample | 0.11 | 0.09 | -0.06 | 0.49 | -0.01 | 0.00 | 0.60 |
| DNA sequences | Mean of within sample pairwise differences | 0.17 | 0.08 | -0.12 | 0.48 | -0.01 | -0.01 | 0.51 |
| DNA sequences | Mean of between sample pairwise differences | 0.00 | 0.00 | 0.03 | 0.51 | -0.01 | -0.01 | 0.54 |
| DNA sequences | *F_ST_* between two samples (Hudson et al., 1992) | -0.51 | -0.21 | 0.46 | 0.06 | -0.01 | 0.00 | 0.03 |
|  |  |  |  |  |  |  |  |  |
|  |  |  |  |  |  |  |  |  |
|  |  |  | 0.6 ≤ |r| | | |  |  |  |
|  |  |  | 0.5 ≤ |r| < 0.6 | | |  |  |  |
|  |  |  | 0.4 ≤ |r| < 0.5 | | |  |  |  |
|  |  |  | 0.3 ≤ |r| < 0.4 | | |  |  |  |
|  |  |  | 0.2 ≤ |r| < 0.3 | | |  |  |  |
|  |  |  | 0.1 ≤ |r| < 0.2 | | |  |  |  |

Summary statistics chosen for rejection-regression step are in cyan cells.

References:

- Chakraborty R and L Jin, 1993. A unified approach to study hypervariable polymorphisms: statistical considerations of determining relatedness and population distances. EXS. 67, 153175.
- Excoffier, L., A. Estoup and J.M. Cornuet, 2005. Bayesian analysis of an admixture model with mutations and arbitrarily linked markers. Genetics 169, 1727-1738.
- Garza JC and E Williamson, 2001. Detection of reduction in population size using data from microsatellite DNA. Mol. Ecol. 10,305-318.
- Goldstein DB, Linares AR, Cavalli-Sforza LL, and Feldman MW, 1995. An evaluation of genetic distances for use with microsatellite loci. Genetics 139, 463-471.
- Hudson,R. R., M. Slatkin and W.P. Maddison, 1992. Estimation of levels of gene flow fom DNA sequence data. Genetics, 132, 583-589.
- Nei M., 1987. Molecular Evolutionary Genetics. Columbia University Press, New York, 512 pp.
- Pascual, M., M.P. Chapuis, F. Mestres, J. Balany_a, R.B. Huey, G.W. Gilchrist, L. Serra and A. Estoup, 2007. Introduction history of *Drosophila subobscura* in the New World: a microsatellite based survey using ABC methods. Mol. Ecol., 16, 3069-3083.
- Rannala, B., and J. L. Mountain, 1997. Detecting immigration by using multilocus genotypes. Pro. Nat. Acad. Sci. USA 94, 9197-9201.
- Tajima, F., 1989. Statistical method for testing the neutral mutationhypothesis by DNA polymorphism. Genetics 123: 585-595
- Weir BS and CC Cockerham, 1984. Estimating F-statistics for the analysis of population structure. Evolution 38: 1358-1370.
