# Supplementary material for: Differential Selection on Carotenoid Biosynthesis Genes as a Function of Gene Position in the Metabolic Pathway: A Study on the Carrot and Dicots
Source: PLoS One. 2012 Jun 18;7(6):e38724. doi: 10.1371/journal.pone.0038724 (PMC3377682; doi:10.1371/journal.pone.0038724)
Supplement: Table S4 — Model checking by comparison of observed dataset and posterior predictive distribution. (DOC) [file pone.0038724.s008.doc]

Table S4. Model checking by comparison of observed dataset and posterior predictive distribution

| **Loci** | **Summary statistics** | **Population** | **Observed value** | **Probability P (simulated<observed)** |  |
| --- | --- | --- | --- | --- | --- |
| Microsatellites | Mean number of alleles across loci | West | 6.18 | 0.321 |  |
| Microsatellites | Mean number of alleles across loci | East | 5.29 | 0.319 |  |
| Microsatellites | Mean gene diversity across loci (Nei, 1987) | West | 0.59 | 0.068 |  |
| Microsatellites | Mean gene diversity across loci (Nei, 1987) | East | 0.60 | 0.143 |  |
| Microsatellites | Mean allele size variance across loci | West | 16.34 | 0.537 |  |
| Microsatellites | Mean allele size variance across loci | East | 21.64 | 0.781 |  |
| Microsatellites | Mean M index across loci (Garza and Williamson, 2001; Excoffier et al., 2005) | West | 0.48 | 0.016 | (*) |
| Microsatellites | Mean M index across loci (Garza and Williamson, 2001; Excoffier et al., 2005) | East | 0.43 | 0.032 | (*) |
| Microsatellites | *F_ST_* between two samples (Weir and Cockerham, 1984) | Both | 0.09 | 0.868 |  |
| Microsatellites | Mean index of classification (1&2) (Rannala and Moutain, 1997; Pascual et al., 2007) | Both | 1.53 | 0.672 |  |
| Microsatellites | Mean index of classification (1&2) (Rannala and Moutain, 1997; Pascual et al., 2007) | Both | 1.66 | 0.901 |  |
| Microsatellites | (δμ )^2^ distance between two samples (Goldstein et al., 1995) | Both | 3.31 | 0.706 |  |
| Microsatellites | Mean number of alleles across loci | Pool | 8.71 | 0.690 |  |
| Microsatellites | Mean gene diversity across loci | Pool | 0.62 | 0.092 |  |
| Microsatellites | Mean allele size variance across loci | Pool | 18.64 | 0.633 |  |
| Microsatellites | Shared allele distance between two samples (Chakraborty and Jin, 1993) | Both | 0.34 | 0.870 |  |
| DNA sequences | Number of distinct haplotypes | West | 6.67 | 0.388 |  |
| DNA sequences | Number of distinct haplotypes | East | 5.33 | 0.465 |  |
| DNA sequences | Number of segregating sites | West | 23.00 | 0.533 |  |
| DNA sequences | Number of segregating sites | East | 14.67 | 0.363 |  |
| DNA sequences | Mean pairwise difference | West | 6.84 | 0.532 |  |
| DNA sequences | Mean pairwise difference | East | 5.31 | 0.413 |  |
| DNA sequences | Variance of the number of pairwise differences | West | 60.63 | 0.631 |  |
| DNA sequences | Variance of the number of pairwise differences | East | 37.66 | 0.517 |  |
| DNA sequences | Tajima's D statistics (Tajima, 1989) | West | 0.51 | 0.564 |  |
| DNA sequences | Tajima's D statistics (Tajima, 1989) | East | 0.18 | 0.317 |  |
| DNA sequences | Number of private segregating sites | West | 12.33 | 0.778 |  |
| DNA sequences | Number of private segregating sites | East | 4.00 | 0.565 |  |
| DNA sequences | Mean of the numbers of the rarest nucleotide at segregating sites | West | 5.64 | 0.736 |  |
| DNA sequences | Mean of the numbers of the rarest nucleotide at segregating sites | East | 3.29 | 0.262 |  |
| DNA sequences | Variance of the numbers of the rarest nucleotide at segregating sites | West | 11.63 | 0.794 |  |
| DNA sequences | Variance of the numbers of the rarest nucleotide at segregating sites | East | 3.17 | 0.377 |  |
| DNA sequences | Number of distinct haplotypes in the pooled sample | Pool | 9.00 | 0.345 |  |
| DNA sequences | Number of segregating sites in the pooled sample | Pool | 27.00 | 0.523 |  |
| DNA sequences | Mean of within sample pairwise differences | Both | 6.40 | 0.492 |  |
| DNA sequences | Mean of between sample pairwise differences | Both | 7.21 | 0.497 |  |
| DNA sequences | *F_ST_* between two samples (Hudson et al., 1992) | Both | 0.11 | 0.632 |  |

This comparison was based on 37 summary statistics. *P* is the proportion of simulated datasets with a corresponding summary statistic lower than that of the observed data set.

References:

- Chakraborty R and L Jin, 1993. A unified approach to study hypervariable polymorphisms: statistical considerations of determining relatedness and population distances. EXS. 67, 153175.
- Excoffier, L., A. Estoup and J.M. Cornuet, 2005. Bayesian analysis of an admixture model with mutations and arbitrarily linked markers. Genetics 169, 1727-1738.
- Garza JC and E Williamson, 2001. Detection of reduction in population size using data from microsatellite DNA. Mol. Ecol. 10,305-318.
- Goldstein DB, Linares AR, Cavalli-Sforza LL, and Feldman MW, 1995. An evaluation of genetic distances for use with microsatellite loci. Genetics 139, 463-471.
- Hudson,R. R., M. Slatkin and W.P. Maddison, 1992. Estimation of levels of gene flow from DNA sequence data. Genetics, 132, 583-589.
- Nei M., 1987. Molecular Evolutionary Genetics. Columbia University Press, New York, 512 pp.
- Pascual, M., M.P. Chapuis, F. Mestres, J. Balany_a, R.B. Huey, G.W. Gilchrist, L. Serra and A. Estoup, 2007. Introduction history of *Drosophila subobscura* in the New World: a microsatellite based survey using ABC methods. Mol. Ecol., 16, 3069-3083.
- Rannala, B., and J. L. Mountain, 1997. Detecting immigration by using multilocus genotypes. Pro. Nat. Acad. Sci. USA 94, 9197-9201.
- Tajima, F., 1989. Statistical method for testing the neutral mutation hypothesis by DNA polymorphism. Genetics 123: 585-595
- Weir BS and CC Cockerham, 1984. Estimating F-statistics for the analysis of population structure. Evolution 38: 1358-1370.
