# Supplementary material for: Differential Selection on Carotenoid Biosynthesis Genes as a Function of Gene Position in the Metabolic Pathway: A Study on the Carrot and Dicots
Source: PLoS One. 2012 Jun 18;7(6):e38724. doi: 10.1371/journal.pone.0038724 (PMC3377682; doi:10.1371/journal.pone.0038724)
Supplement: Table S5 — Accession number of genes used for the phylogenetic analysis. (DOC) [file pone.0038724.s009.doc]

Table S5. Accession number of genes used for the phylogenetic analysis

|  | ***IPI*** | ***PDS*** | ***CRTISO*** | ***LCYB*** | ***LCYE*** | ***CHXE*** | ***ZEP*** |
| --- | --- | --- | --- | --- | --- | --- | --- |
| ***Arabidopsis lyrata*** | XM_002884293.1 | XM_002868257.1 | XM_002892314.1 | XM_002884738.1 | XM_002864447.1 | XM_002876144.1 | XM_002864986.1 |
| ***Arabidopsis thaliana*** | NM_111146.3 | AY040007.1 | NM_100559.3 | NM_111858.2 | AY079371.1 | AY424805.1 | NM_180954.2 |
| ***Daucus carota*** | DQ192183.1 | DQ222429.1 | DQ192188.1 | DQ192190.1 | DQ192192.1 | DQ192196.1 | DQ192197.1 |
| ***Populus trichocarpa*** | XM_002325433.1 | XM_002321068.1 | XM_002323326.1 | XM_002308867.1 | POPTR_0006s14920.1^a^ | XM_002326353.1 | XM_002307229.1 |
| ***Ricinus communis*** | XM_002514802.1 | XM_002518582.1 | XM_002524053.1 | XM_002531452.1 | XM_002514090.1 | XM_002519381.1 | XM_002523541.1 |
| ***Solanum lycopersicum*** | AK328287.1 | S36691.1 | AK247093.1 | AK319553.1 | AK321362.1 | BT012891.1 | AK327384.1 |
| ***Vitis vinifera*** | XM_002277899.1 | XM_002264231.1 | XM_002269518.1 | XM_002275733.1 | XM_002281128.1 | XM_002264979.1 | AY337615.1 |

^a^ Retrieved from http://phytozome.net
