# Supplementary material for: Differential Selection on Carotenoid Biosynthesis Genes as a Function of Gene Position in the Metabolic Pathway: A Study on the Carrot and Dicots
Source: PLoS One. 2012 Jun 18;7(6):e38724. doi: 10.1371/journal.pone.0038724 (PMC3377682; doi:10.1371/journal.pone.0038724)
Supplement: Text S1 — Construction and validation of the divergence model, and neutral expectations. (DOCX) [file pone.0038724.s010.docx]

**Text S1. Construction and validation of the divergence model, and neutral expectations**

**Construction of a model integrating population divergence in the cultivated carrot**

A previous analysis of the current sample using microsatellite loci showed individuals clustered in two groups matching their geographical origin, called ‘Western’ and ‘Eastern’ groups [1]. In order to take this population subdivision into account in neutrality tests, we built a demographic model using approximate Bayesian computation (ABC) [2] in which two populations diverged from an ancestral population (divergence model) and used it to test carotenoid biosynthesis genes for signatures of selection.

Posteriors obtained after a rejection-regression step are shown in Figure S1. The population effective size (*N_w_*) was estimated at 4027 individuals (95% confidence interval (CI): 1948 to 35362) for the Western population (*N_w_*) and at 2748 individuals (95% CI: 1327 to 27280) for the Eastern population (*N_e_*). This is consistent with lower diversity (*H*=0.603 for microsatellites and *π*=7.72×10^-3^ for the three anonymous loci) in the Eastern population than in the Western population (*H*=0.627 for microsatellites and *π*=9.59×10^-3^ for the three anonymous loci). The population effective size of the ancestral population (*N_a_*) was estimated at 51217 individuals (95% CI: 24140 to 190768). The divergence time (*T_d_*) between Western and Eastern populations was dated 516 generations ago (95% CI: 368 to 638), i.e. 1032 years ago (95% CI: 726 to 1276). The average microsatellite loci mutation rate *µ_ssr_* is 4.9×10^-5^ mutations per generation (95% CI: 1.8×10^-5^ to 1.9×10^-4^). Out of 17 microsatellites, 14 have dinucleotide repeat motifs, one has trinucleotide repeat motifs and two have tetranucleotide repeat motifs. For the purpose of comparison, in maize, the mutation rate per generation is estimated at 7.7×10^-4^ (95% CI: 5.2×10^-4^ to 1.1×10^-3^) and in durum wheat at 2.4×10^-4^ (95% CI: 1.4×10^-4^ to 4.2×10^-4^) for microsatellites with dinucleotide repeat motifs [3,4]. The parameter of the geometric distribution *P_SSR_* was estimated at 0.37 (95% CI: 0.14 to 4.28). The DNA sequence mutation rate *µ_seq_* was estimated at 4.66×10^-8^ mutations per site per generation (95% CI: 1.81×10^-8^ to 2.30×10^-7^). This estimation is in agreement with the nucleotide substitution rate in maize estimated at 2.9×10^-8^ or 3.3×10^-8^ substitutions per site per generation [5].

In order to check the divergence model, the observed data, the prior distribution, and the posterior predictive distribution were represented in a principal component analysis in the space of summary statistics (Figure S2). In the figure, the posterior predictive distribution is located at the center of prior distribution, indicating that priors were large enough in the analysis. Observed data are located at the center of the posterior predictive distribution, implying the rejection-regression step was correct. Twenty-seven summary statistics calculated on the observed dataset were then ranked against the distribution of the corresponding summary statistics from the posterior predictive distribution (Table S3). Only the mean M index across loci in each population differed significantly between the observed and the posterior predictive distribution, indicating significantly lower M values for posterior predictive distribution than observed data [6]. Therefore we considered our model to be suitable for testing signatures of selection on carotenoid biosynthesis genes.

**Neutral expectations under the divergence model**

According to sequence length and to the number of sequences of the seven carotenoid biosynthesis genes, seven datasets were generated by coalescent simulations using combinations of parameters sampled from posterior distribution. The rate of undetected back mutations in the outgroup branch, and therefore the rate of misorientations when assigning ancestral alleles, was estimated at *P_M_*=0.06 [7]. This rate was included in coalescent simulations by generating a similar bias in simulated datasets.

Tajima’s *D* in the pooled dataset under the divergence model deviated slightly toward positive values (mean= 0.11; 95% CI: -1.54 to 2.12) (Figure S3). This means that there is a weak excess of intermediate frequency alleles at the subspecies level, which would be consistent with the moderate population structure detected between Western carrots and Eastern carrots [1]. However, this deviation towards positive values for Tajima’s *D* was slightly reinforced within each population: Western (mean=0.2; 95% CI: -1.59 to 2.14) and Eastern (mean=0.27; 95% CI: -1.67 to 2.17). Deviations towards slightly positive normalized Fay and Wu’s *H* were found in the three following samples: pooled sample (mean=0.35, lower bound of 95% CI=-1.9), Western group (mean=0.33, lower bound of 95% CI=-2), Eastern group (mean=0.32, lower bound of 95% CI=-1.95) (Figure S3). This attests to a slight excess of intermediate frequency derived alleles, consistent with the results of Tajima’s *D*. With the divergence model, *F_ST_* between Western and Eastern groups decreased when *θ_w_* increased (Figure S4). This result is congruent with a recent work showing that *F_ST_* tends to zero when the mutation rate increases [8]. Average *θ_w_* per gene was 8.5 among seven carotenoid biosynthesis genes [1]. For *θ_w_* =8.5 ±1.5, the mean *F_ST_* expectation under the divergence model was 0.034 (95% CI: -0.029 to 0.253).

**Bibliography of Text S1**

1. Clotault J, Geoffriau E, Lionneton E, Briard M, Peltier D (2010) Carotenoid biosynthesis genes provide evidence of geographical subdivision and extensive linkage disequilibrium in the carrot. Theor Appl Genet 121: 659–667.

2. Beaumont MA, Zhang W, Balding DJ (2002) Approximate Bayesian Computation in population genetics. Genetics 162: 2025–2035.

3. Thuillet A-C, Bru D, David J, Roumet P, Santoni S, et al. (2002) Direct estimation of mutation rate for 10 microsatellite loci in Durum Wheat, *Triticum turgidum* (L.) Thell. ssp *durum* desf. Mol Biol Evol 19: 122–125.

4. Vigouroux Y, Jaqueth JS, Matsuoka Y, Smith OS, Beavis WD, et al. (2002) Rate and pattern of mutation at microsatellite loci in maize. Mol Biol Evol 19: 1251–1260.

5. Clark RM, Tavaré S, Doebley J (2005) Estimating a nucleotide substitution rate for maize from polymorphism at a major domestication locus. Mol Biol Evol 22: 2304–2312.

6. Garza JC, Williamson EG (2001) Detection of reduction in population size using data from microsatellite loci. Mol Ecol 10: 305–318.

7. Baudry E, Depaulis F (2003) Effect of misoriented sites on neutrality tests with outgroup. Genetics 165: 1619–1622.

8. Kronholm I, Loudet O, de Meaux J (2010) Influence of mutation rate on estimators of genetic differentiation - lessons from *Arabidopsis thaliana*. BMC Genet 11: 33.
